# Dogs Leaving the ICU Carry a Very Large Multi-Drug Resistant Enterococcal Population with Capacity for Biofilm Formation and Horizontal Gene Transfer

**DOI:** 10.1371/journal.pone.0022451

**Published:** 2011-07-19

**Authors:** Anuradha Ghosh, Scot E. Dowd, Ludek Zurek

**Affiliations:** 1 Department of Diagnostic Medicine and Pathobiology, College of Veterinary Medicine, Kansas State University, Manhattan, Kansas, United States of America; 2 Medical Biofilm Research Institute, Lubbock, Texas, United States of America; 3 Department of Entomology, Kansas State University, Manhattan, Kansas, United States of America; University of Birmingham, United Kingdom

## Abstract

The enterococcal community from feces of seven dogs treated with antibiotics for 2–9 days in the veterinary intensive care unit (ICU) was characterized. Both, culture-based approach and culture-independent 16S rDNA amplicon 454 pyrosequencing, revealed an abnormally large enterococcal community: 1.4±0.8×10^8^ CFU gram^−1^ of feces and 48.9±11.5% of the total 16,228 sequences, respectively. The diversity of the overall microbial community was very low which likely reflects a high selective antibiotic pressure. The enterococcal diversity based on 210 isolates was also low as represented by *Enterococcus faecium* (54.6%) and *Enterococcus faecalis* (45.4%). *E. faecium* was frequently resistant to enrofloxacin (97.3%), ampicillin (96.5%), tetracycline (84.1%), doxycycline (60.2%), erythromycin (53.1%), gentamicin (48.7%), streptomycin (42.5%), and nitrofurantoin (26.5%). In *E. faecalis*, resistance was common to tetracycline (59.6%), erythromycin (56.4%), doxycycline (53.2%), and enrofloxacin (31.9%). No resistance was detected to vancomycin, tigecycline, linezolid, and quinupristin/dalfopristin in either species. Many isolates carried virulence traits including gelatinase, aggregation substance, cytolysin, and enterococcal surface protein. All *E. faecalis* strains were biofilm formers *in vitro* and this phenotype correlated with the presence of *gelE* and/or *esp*. *In vitro* intra-species conjugation assays demonstrated that *E. faecium* were capable of transferring tetracycline, doxycycline, streptomycin, gentamicin, and erythromycin resistance traits to human clinical strains. Multi-locus variable number tandem repeat analysis (MLVA) and pulsed-field gel electrophoresis (PFGE) of *E. faecium* strains showed very low genotypic diversity. Interestingly, three *E. faecium* clones were shared among four dogs suggesting their nosocomial origin. Furthermore, multi-locus sequence typing (MLST) of nine representative MLVA types revealed that six sequence types (STs) originating from five dogs were identical or closely related to STs of human clinical isolates and isolates from hospital outbreaks. It is recommended to restrict close physical contact between pets released from the ICU and their owners to avoid potential health risks.

## Introduction

National and international surveillance programs on antimicrobial resistance such as SENTRY, SCOPE, SWEDRES, SVARM, FAO, DANMAP, and NARMS have been established for people as well as food animals in many parts of the world. Although recommended, pet animals are typically not included in such programs [1], [2]. According to the American Veterinary Medical Association, there are about 72 million dogs and 81 million cats in American households (http://www.avma.org). Unfortunately, no comprehensive data are available on consumption of antimicrobials in small animal veterinary practices in the USA. In recent past, pet animals have been considered as a putative reservoir of antimicrobial resistant bacteria based on sporadic cases showing transmission of pathogenic bacterial strains such as *Staphylococcus aureus* [3], *S. intermedius* [4], *S. pseudintermedius* [5], *Campylobacter jejuni* [6], and *Enterococcus faecium* [7] between pets and owners. Furthermore, shedding of high concentrations of *Clostridium perfringens*, *E. faecalis* and *E. faecium* in feces of dogs with diarrhea was reported by Bell *et al.* [8]. The Centers for Disease Control and Prevention has stated that immunocompromised groups and children may be at risk for infections with canine zoonotic agents (www.cdc.gov/healthypets/animals/dogs.html).

Enterococci, ubiquitous in nature and a common commensal of the intestinal microbiota of people and animals [9], have emerged as pathogens that rank third among nosocomial infections due to their resistance to antibiotics, putative virulence traits, and their biofilm forming capacity [10], [11]. The threat posed by enterococci is magnified due to their ability to horizontally transfer antibiotic resistance and virulence determinants to other bacteria [12], [13]. Only a very few studies addressed the enterococcal population in pets and all of these focused on healthy animals. In Europe, Damborg *et al.* [14], [15] reported wide occurrence of human hospital-associated enterococcal clones among dogs. In the USA, only one research group examined the dogs and cats as a potential source of antibiotic resistant enterococci [16]. In the subsequent study, they also determined the mechanism of antibiotic resistance and assessed the clonality of the isolates [17]. However, companion animals under antibiotic treatments have so far been mostly neglected from the perspective of studying antibiotic resistant microbiota, nosocomial strains, and potential animal and public health implications. Within the hospital environment, intensive care unit (ICU) provides the most ambient condition for survival of enterococci as they can withstand an extensive use of antibiotics and disinfectants [18], [19], [20].

We hypothesized that companion animals (dogs) treated with antibiotics in the ICU become a reservoir of antibiotic resistant and potentially virulent enterococcal population and the corresponding resistance traits are horizontally transferrable. The goal of this study was to characterize enterococci isolated from the feces of dogs from the ICU in order to evaluate their potential for nosocomial and zoonotic infections. In addition, we assessed the diversity of the overall fecal bacterial community of these dogs by 16S rRNA gene-based 454 pyrosequencing.

## Methods

### Ethics statement

The ethics permit from the Institutional Review Board was not required. Collection of canine fecal samples did not involve any direct contact with animals. The human blood for hemolysis detection was purchased directly from Rockland Immunochemicals Inc. (Gilberstville, PA) and was used based on manufacturer's instructions.

### Sample collection, isolation, and identification of enterococci

During 2008–09, over a period of four months, fresh feces of seven dogs were sampled after a stay at the ICU of the Veterinary Medicine Teaching Hospital (Kansas State University) for 2–9 days on an antibiotic treatment. Disease history and treatments of the dogs in the ICU are shown in Table S1. One gram of feces was resuspended in 10 ml of phosphate buffered saline and up to 30 presumptive enterococcal colonies were randomly selected from each sample following the standard protocol [21]. The concentration of enterococci was calculated in CFU g^−1^ of feces and the isolates were identified to the genus and species levels following methods described previously [21].

### Assessment of the overall fecal bacterial diversity by 454 pyrosequencing

Total genomic DNA was extracted from the same fecal samples (0.5 g) as above using FastDNA® SPIN kit for soil (MP Biomedicals) following manufacturer's instructions. The bacterial tag-encoded FLX 16S rDNA amplicon parallel pyrosequencing and post sequencing processing were carried out at the Medical Biofilm Research Institute (Lubbock, TX) as described by Dowd *et al.* [22] and Middelbos *et al.* [23]. Data were analyzed and interpreted using Sequencher 4.8 (Gene Codes) for alignment and sequence editing, MOTHUR [24] for diversity and richness, and Blast2GO for the NCBI GenBank search.

### Antibiotic susceptibility testing and transfer of antibiotic resistance traits

Antibiotic sensitivity was determined by the disc diffusion method on Mueller-Hinton agar (Difco) using 10 different antibiotics (µg disc^−1^): ampicillin (10), tetracycline (30), doxycycline (30), gentamicin (120), erythromycin (15), enrofloxacin (5), vancomycin (30), quinupristin/dalfopristin (15), nitrofurantoin (300), and tigecycline (15). Resistance to streptomycin (2,000 µg ml^−1^) and linezolid (8 µg ml^−1^) was assessed by agar dilution technique on brain heart infusion (BHI) (BBL) agar. Minimum inhibitory concentration (MIC, µg ml^−1^) was determined for a subset of *E. faecium* strains (resistant to 4–6 antibiotics and further used for conjugation assays) by broth microdilution technique using Mueller-Hinton broth (BBL). The results were interpreted according to the guidelines of the Clinical and Laboratory Standards Institute [25], [26]. Routine quality control of antibiotic discs was performed using control strains of *E. faecalis* ATCC 19433 and *E. faecium* ATCC 19434. Multi-drug resistance was defined as resistance to three or more antibiotics, regardless of class.

Broth and filter mating experiments were carried out as described by Ike *et al.* [27] and Tendolkar *et al.* [28], respectively, to study the mobility of seven antibiotic resistance traits from multi-drug resistant *E. faecium* strains (6–8 isolates for each trait) to *E. faecium* clinical strains. The recipients included the following strains with appropriate markers: TX5034 (spectinomycin, MIC = 250 µg ml^−1^) [29] for tetracycline and doxycycline; TX1330 (rifampicin, MIC = 24 µg ml^−1^) [30] for ampicillin; ATCC 51559 (rifampicin, MIC = 24 µg ml^−1^) [31] for streptomycin and enrofloxacin; 45–24 (linezolid, MIC = 8 µg ml^−1^) [32] for erythromycin; and 38–42 (linezolid, MIC = 8 µg ml^−1^) [32] for gentamicin. Both assays were performed with a donor and recipient ratio of 1∶10. After allowing mating for 4 h in broth and 16 h on filter, the mixed culture was dilution plated on to BHI agar supplemented with suitable combinations of antibiotics and incubated for 24–48 h at 37°C. The transfer frequency for each isolate was calculated as the number of transconjugants per donor CFU. The transconjugants were examined for the phenotypic expression of the resistance traits by determination of MICs as mentioned above.

### Genotypic and phenotypic characterization of virulence traits

Multiplex PCR was performed to screen the identified isolates for four putative virulence determinants: *gelE* (gelatinase), *cylA* (cytolysin), *asa1* (aggregation substance), and *esp* (enterococcal surface protein) [33]. These isolates were also tested for gelatinase (protease) activity on Todd Hewitt agar (BBL) with 1.5% skim milk, expression of the *asa1* gene (only in *E. faecalis*) using clumping assay, and cytolysin expression by *β*-hemolysis on Columbia blood agar base (Difco) with 5.0% human blood (Rockland Immunochemicals) as described previously [34].

### Biofilm assay on polystyrene microtiter plates

Strains were inoculated in M17 broth (Oxoid) in polystyrene round-bottomed 96 well plates (Corning) for bacterial growth and biofilm formation as described previously [35]. Biofilm was quantified using crystal violet staining method as described by Hancock and Perego [36]. *E. faecalis* V583 was used as the positive control.

### Genotyping by multi-locus variable number tandem repeat analysis (MLVA)

MLVA typing was used to assess the clonality of all 112 multi-drug resistant *E. faecium* (with exception of one isolate from dog ICU-6 that was not viable) according to the protocol described by Top *et al.* [37], with the following modifications. In all cases, template DNA was obtained from freshly boiled cells in distilled water and the initial denaturation was 94°C for 4 min. For amplification of VNTR-2, reaction was carried out in 25 µl with 0.2 mM MgCl_2_ at an annealing temperature of 65°C. MLVA profiles were submitted to the MLVA database (http://www.umcutrecht.nl/subsite/MLVA/) and assigned their MLVA type (MT). Clustering of MTs was performed using the eBURST ver. 3 algorithm implemented as a Java applet at http://eburst.mlst.net described by Feil *et al.* [38]. eBURST clustering displayed all MTs from a large MLVA database in a single diagram as a snapshot of *E. faecium* clonal diversity and the MTs were further described as single/double/triple-locus variants (SLVs, DLVs, TLVs).

### Genotyping by pulsed-field gel electrophoresis (PFGE)

In order to confirm the MLVA clustering a subset of 49 *E. faecium* was typed by PFGE following the protocol of Amachawadi *et al.* [39] with minor modifications. Agarose plugs were restriction digested with 40 U of *Apa*I (Promega) for 4 h at 37°C. The digested plugs were run on to a 1.0% SeaKem Gold Agarose (Lonza) gel using CHEF Mapper (Bio-Rad) with initial pulse time for 1 s and final time for 20 s at 200 V for 21 h. Cluster analysis was performed with BioNumerics (Applied Maths) by using the band-based Dice correlation coefficient and the unweighted pair group mathematical average algorithm (UPGMA). *E. faecium* ATCC 19434 was used as the reference strain.

### Genotyping by multi-locus sequence typing (MLST)

One representative of each of nine MTs of *E. faecium* was typed using MLST. Seven loci were PCR amplified according to the standard protocol (http://efaecium.mlst.net/misc/info.asp) using Maxima Hot Start PCR Master Mix (Fermentas Inc.). PCR products were purified using DNA Clean and Concentrator kit (Zymo Research Corp.). Both strands were sequenced by Applied Biosystems 3730 DNA Analyzer using the same primers. Sequences were edited, aligned and compared to the reference set of alleles using CodonCode Aligner ver. 2.0.4. MLST profiles were submitted to the MLST database (http://efaecium.mlst.net) and assigned their MLST type (ST). eBURST clustering was performed as described above using the entire *E. faecium* MLST database.

## Results

### Overall bacterial diversity and enterococcal concentration in feces of the ICU dogs

Dogs in this study were of diverse breeds covering small to large size and a broad age group from 2 months to 14 year old (Table S1). In the ICU, they were treated for various diseases with antibiotics including *β*-lactams, tetracycline, fluoroquinolone, and a third generation cephalosporin (Table S1).

**Figure 1:**
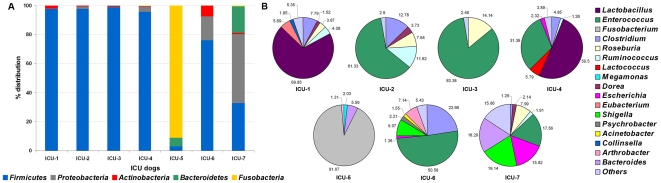
Fecal bacterial diversity in ICU dogs. Diversity based on 16S rDNA amplicon 454 pyrosequencing at (A) phylum level; and (B) genus level (numbers = % prevalence; Others = genera contributing <1% of the total population). ICU = intensive care unit.

The overall fecal bacterial diversity on the phylum level based on pyrosequencing results is shown in Figure 1A. *Firmicutes* represented the dominant (76.0–98.9%) phylum in 5 out of 7 dogs, followed by *Fusobacteria* in the dog ICU-5 (91.1%). Dog ICU-7 had relatively even distribution among *Firmicutes* (33.7%), *Proteobacteria* (47.7%), and *Bacteroidetes* (18.5%). *Proteobacteria* constituted relatively large portion of the bacterial community in two dogs (16.5% in ICU-6 and 47.7% in ICU-7). Members of the *Bacteroidetes* phylum were detected in the dogs ICU-5 (6.1%) and ICU-7 (18.5%). Presence of *Actinobacteria* was rare with the exception of dog ICU-6 (7.3%) (Figure 1A).

On the genus level, 17 different genera were detected (cut off: ≥1.0% of all sequences per sample) (Figure 1B). Five out of 7 dogs had abnormally high population of enterococci (range: 17.6–83.4%). *Lactobacillus* was dominant (60.2±9.7%) in the dogs ICU-1 and ICU-4; *Fusobacterium* was very high (91.1%) in the 2 month old puppy ICU-5. Genera including *Clostridium*, *Dorea*, *Roseburia*, *Ruminococcus*, and *Megamonas* were detectable in most samples. Members of *Enterobacteriaceae* (*Escherichia*, *Shigella*) were found in three dogs (ICU-4, 6 and 7) while *Moraxellacaea* (*Psychrobacter* and *Acinetobacter*) was abundant only in the dog ICU-5 (Figure 1B).

Bacterial species richness in the canine feces derived from 2,318±170 bp good quality sequences per sample is depicted in Table S2. Overall, at the distance of 0.03 (species level), 52.4±8.9 operational taxonomic units (OTU) were detected with the Shannon diversity index (H′) 1.6±0.3. The non-parametric estimators Chao1 and ACE (abundance-based coverage estimator) project a range of 61.6±11.9 to 68.5±13.4 OTUs at the 0.03 distance level. The corresponding rarefaction values (Rf) ranged from 18.2 to 85.9 at the species level (Table S2). Table S3 illustrates in details the bacterial diversity at the rank of species.

Culture-dependent technique confirmed that the enterococcal concentration in all samples was very high with a mean of 1.4±0.8×10^8^ CFU g^−1^ feces. On the other hand, the enterococcal community was comprised of only two species *E. faecalis* and *E. faecium*, and 3 out of 7 dogs carried only one species (Table 1).

**Table 1:** Enterococcal concentration and species diversity in the feces of dogs from the intensive care unit (ICU).

Sample ID.	Concentration (CFU g^−1^)	Diversity [n* (%)]	
		*E. faecalis*	*E. faecium*
ICU-1	8.3×10^4^	30 (100)	0
ICU-2	2.2×10^8^	10 (34.4)	20# (65.5)
ICU-3	1.2×10^8^	12 (40.0)	18 (60.0)
ICU-4	1.4×10^8^	25 (83.3)	5# (16.7)
ICU-5	4.6×10^5^	0	30# (100)
ICU-6	5.8×10^7^	0	30 (100)
ICU-7	1.3×10^8^	17 (56.6)	13 (43.4)

*n = number of isolates from each sample.

# one isolate lost during sub-culturing and not analyzed further.

### Antibiotic susceptibility and intra-species conjugal transfer of antibiotic resistance traits

**Figure 2:**
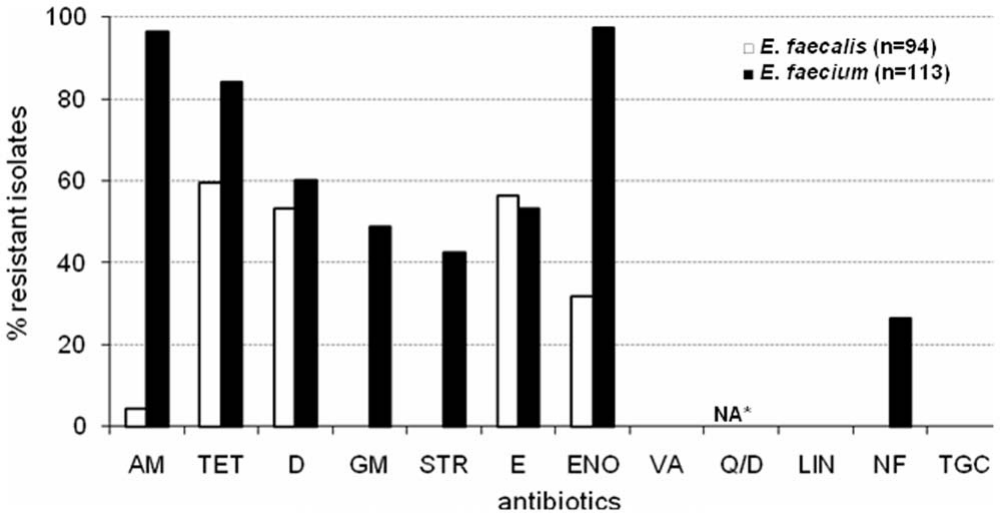
Antibiotic resistance profile of enterococci from the feces of ICU dogs. AM = ampicillin, TET = tetracycline, D = doxycycline, GM = gentamicin, STR = streptomycin, E = erythromycin, ENO = enrofloxacin, VA = vancomycin, Q/D = quinupristin/dalfopristin, LIN = linezolid, NF = nitrofurantoin, TGC = tigecycline, ICU = intensive care unit. *not applicable for *E. faecalis* isolates due to their intrinsic resistance.

All isolates were screened for their susceptibility to 12 antibiotics representing 10 classes. The choice of antibiotics was primarily based on the drugs commonly used to treat human enterococcal infections and also frequently used in veterinary medicine. *E. faecium* was very frequently (43.4%) multi-drug (6–8 antibiotics) resistant with the most common (22.1%) resistance combination of ampicillin, tetracycline, doxycycline, gentamicin, erythromycin, and enrofloxacin (Table S4). The majority (>80%) of *E. faecium* showed resistance to wide spectrum of antibiotics including fluoroquinolone (enrofloxacin: 97.3%), *β*-lactam (ampicillin: 96.5%), and tetracyclines (tetracycline: 84.1%; doxycycline: 60.2%), followed by resistance to macrolide (erythromycin: 53.1%), aminoglycosides (gentamicin: 48.7%; streptomycin: 42.5%), and nitrofurantoin (26.5%) (Figure 2). The MICs determined for a subset of multi-drug resistant *E. faecium* was high and in the MIC range of human clinical *E. faecium* isolates (Table 2). In addition, a considerable number of *E. faecalis* (44.8%) was multi-drug (3–4 antibiotics) resistant with tetracycline, doxycycline, and erythromycin or enrofloxacin as the most common combinations (Table S4). These strains were frequently resistant to tetracycline (59.6%), erythromycin (56.4%), doxycycline (53.2%), and enrofloxacin (31.9%) (Figure 2). The antibiotic resistance profile for each isolate from individual ICU dogs is presented in Table S5.

**Table 2:** Intra-species horizontal transfer of antibiotic resistance traits in multi-drug resistant *E. faecium* isolated from the feces of dogs from the intensive care unit (ICU) to *E. faecium* clinical isolates.

Antibiotic resistance trait		Donor	Broth mating (4 h)		Filter mating (16 h)		Transconjugant	Recipient
	n*	^MIC range^ (µg ml^−1^)	% transferred	Transfer rate† (T/D*)	% transferred	Transfer rate† (T/D*)	^MIC range^ (µg ml^−1^)	^MIC range^ (µg ml^−1^)
Tetracycline	6	32–64	100	7.4±3.3×10^−5^	100	3.3±2.4×10^−4^	16–32	<1
Doxycycline	6	0.3–0.6	100	3.7±1.8×10^−4^	100	4.6±2.6×10^−4^	0.3–0.6	<1
Ampicillin	8	32–64	0	0	0	0	-	<1
Streptomycin	6	>4000	0	0	100	8.6±4.1×10^−7^	2000–4000	64
Gentamicin	6	>1000	50	2.6±1.1×10^−3^	83	2.2±1.5×10^−3^	>1000	16
Erythromycin	6	32–64	17	1.2×10^−7^	100	3.4±1.9×10^−5^	16–64	<2
Enrofloxacin	6	16–32	0	0	0	0	-	<1

* n = number of isolates tested; D = donor; T = transconjugants.

MIC = minimum inhibitory concentration.

† values presented as mean ± SEM.

The multi-drug resistant *E. faecium* strains were further examined for the potential of horizontal gene transfer by broth and filter conjugation assays. All *E. faecium* isolates tested transferred traits conferring resistance to tetracycline and doxycycline to a clinical strain of *E. faecium* in broth as well as filter mating with a transfer rate of 10^−4^ to 10^−5^ transconjugants per donor (T/D) (Table 2). For streptomycin resistance, transconjugants were obtained only in filter mating with a low rate of 10^−7^ T/D. On the other hand, the gentamicin resistance trait was transferred at a high frequency (10^−3^ T/D) from 3 out of 6 isolates in broth mating and 5 out 6 isolates in filter mating (Table 2). The transferability of erythromycin resistance was higher in filter mating (10^−5^ T/D) where transconjugants were obtained from all six isolates in contrast to broth mating where only 1 out of 6 isolates could transfer the trait with a lower transfer rate (10^−7^ T/D). None of the tested isolates transferred ampicillin and enrofloxacin resistance traits. The transconjugants obtained were phenotypically confirmed by comparing their MICs for appropriate antibiotics with respect to that of the donor and recipient strains (Table 2). The conjugation results were further supported by PFGE analysis where the genotypes of transconjugants, donors and recipients were compared (data not shown).

### Virulence factors and biofilm formation

**Figure 3:**
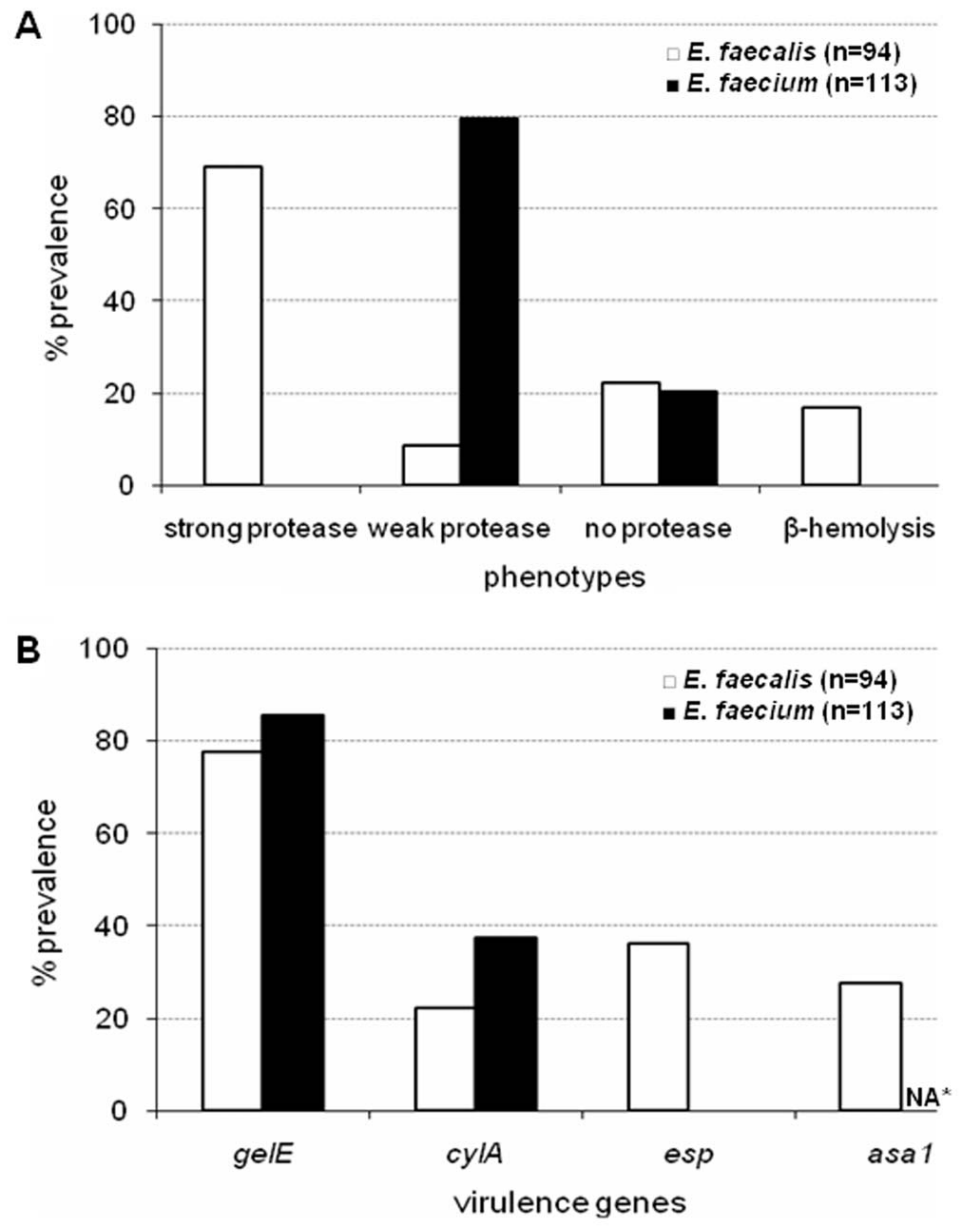
Profiles for virulence traits of enterococci from the feces of ICU dogs. Profiles for (A) gelatinase (protease) and hemolysis phenotypic assays, and (B) presence of virulence genes. ICU = intensive care unit. *not applicable for *E. faecium*.

In *E. faecalis*, the *gelE* gene was detected frequently (73/94, 77.6%) and 89.0% (65/73) of those with *gelE* showed strong gelatinase activity while the rest were weakly gelatinolytic (Figures 3A and B). In contrast, although *E. faecium* also commonly carried *gelE* (97/113, 85.8%), the majority of these (90/97, 92.7%) exhibited only weak gelatinase activity (Figures 3A and B). The enterococcal surface protein gene (*esp*) was detected in *E. faecalis* (34/94, 36.2%) only (Figure 3B). None of the *E. faecalis* was positive for the aggregation substance by phenotype (clumping assay) although 27.6% (26/94) of them carried *asa1*. The *cylA* gene was detected in *E. faecalis* (21/94, 22.3%) and most of these strains were *β*-hemolytic on human blood. In contrast, none of the *E. faecium* positive for *cylA* (42/113, 37.2%) was *β*-hemolytic (Figures 3A and B). The virulence genotypic profile for each ICU dog isolate is illustrated in Table S5.

**Figure 4:**
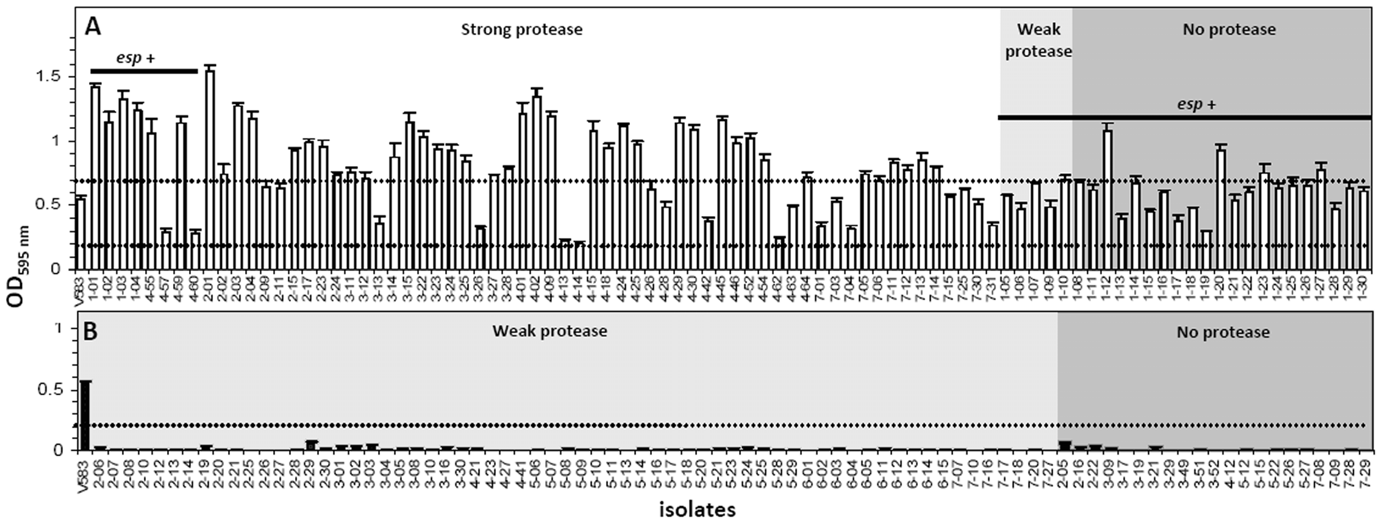
Biofilm formation, gelatinase (protease) phenotype and presence of *esp* in enterococci from the feces of ICU dogs. (A) *E. faecalis* (n = 90), (B) *E. faecium* (n = 85). The dashed lines indicate biofilm formation activity (<0.2 = no biofilm, 0.2–0.7 = biofilm, >0.7 = strong biofilm). Bars correspond to the mean ± SEM of 5 replicates. *E. faecalis* V583 used as a positive control.

All *E. faecalis* isolates tested were biofilm formers (OD_595_>0.2) and many of them (47/90, 52.2%) produced a strong biofilm (OD_595_>0.7) (Figure 4A). Overall, biofilm formation correlated with the presence of strong gelatinase phenotype and/or with the *esp* gene. In contrast, none of *E. faecium* formed biofilm and all of them lacked the strong gelatinase phenotype as well as *esp* (Figure 4B).

### Clonal analysis and relatedness of *E. faecium*

**Figure 5:**
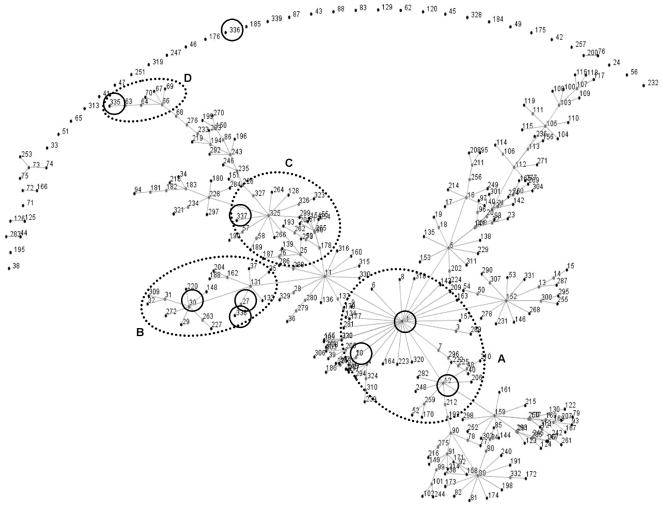
Clustering of nine *E. faecium* MTs from the feces of ICU dogs. eBURST clustering of nine multi-locus variable number tandem repeat analysis types (MTs) representing 109 *E. faecium* isolates from the present study (indicated by solid line circles), with one representative isolate from each of 339 MTs available in the MLVA database. Each MT is represented as a node and differs in one VNTR locus. Dotted line circles indicate MTs from A: clinical infections, hospital outbreaks, hospital and community surveys; B: clinical infections, hospital environment; C: clinical infections, animals and birds (ostrich, chicken, dog, pig); D: calves and community survey. ICU = intensive care unit.

Clonal analysis based on MLVA assigned 109 *E. faecium* isolates (3 strains from dog ICU-4 were not typeable) to nine MTs including four novel ones (MTs 335, 336, 337 and 338) (Figure 5). The population snapshot of the entire *E. faecium* MLVA database generated by eBURST offered a view of all major and minor clonal complexes and indicated MT-1 corresponding to the clonal complex involved in hospital acquired infections (CC-1) as the primary founder (Figure 5). MTs 10 and 12 (SLVs of MT-1) were directly related to MT-1 and included isolates from hospital outbreaks, clinical infections, and also from hospital and community surveys (http://www.umcutrecht.nl/subsite/MLVA/). Another three MTs: MT-27 (TLV of MT-1, and DLV of MT-12), MT-30 (TLV of MT-1, DLV of MT-10, and SLV of MT-27), and MT-338 (DLV of MT-27 and MT-30) clustered together and were closely related to isolates from clinical infections and hospital environment. MT-337 (DLV of MT-30, and SLV of MT-27) grouped with human clinical isolates along with isolates from various animals including ostrich, chicken, dog, and pig. MT-336 (4-locus variant of MT-1) distantly placed as an individual MT not linked to any other MT in the database whereas MT-335 (differed in all 6 loci from MT-1 and it was TLV of MT-336) was on the same branch with calf isolates (MTs 64, 66, 67, 69 and 70) (Figure 5). Interestingly, none of the MTs from our ICU dogs except MT-337 showed close association with the MTs described previously from dogs (MTs 53, 60 and 124) (http://www.umcutrecht.nl/subsite/MLVA/).

**Figure 6:**
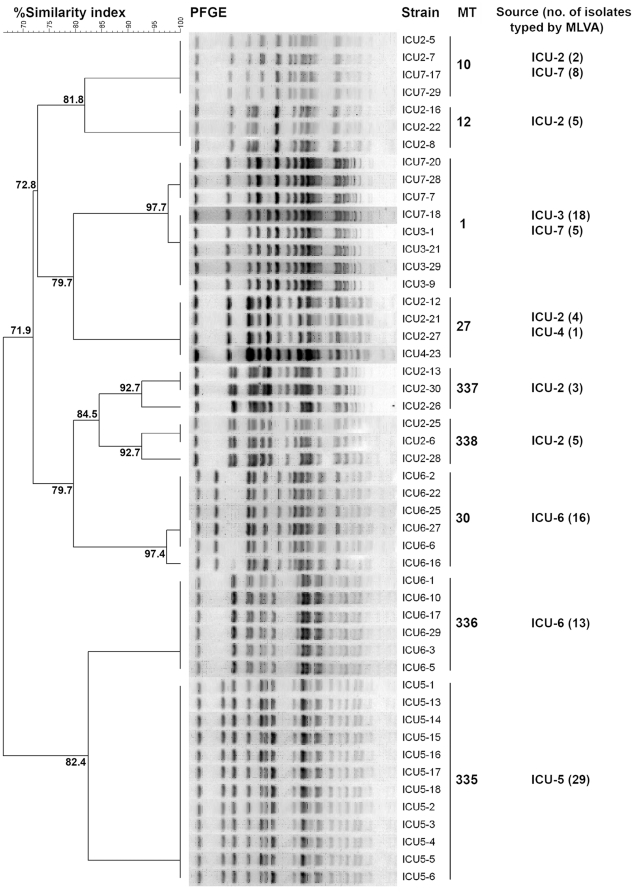
Clonality of *E. faecium* from the feces of ICU dogs based on PFGE. Dendrogram based on *Apa*I restriction pattern resolved by pulsed-field gel electrophoresis (PFGE) depicting the relationships of 49 representative *E. faecium* strains isolated from dogs from the intensive care unit (ICU). The scale indicates the level of pattern similarity. Strain numbers and their association with respective MLVA types (MTs) are indicated.

Clonality assessment based on PFGE and its correlation to MLVA is shown in Figure 6. Dogs ICU-3 and ICU-5 were monoclonal whereas dogs ICU-6 and ICU-7 had only two clonal populations. Dog ICU-2 was the oldest one and harbored the most diverse enterococcal population with 5 MTs corresponding to 5 pulsotypes. In dog ICU-4, only one of five isolates was viable and typed. Interestingly, three identical MTs (MTs 1, 10 and 27) were shared among dogs ICU-2, ICU-3, ICU-4, and ICU-7 and this was supported by PFGE clustering as well. Overall, PFGE dendrogram based on a subset population (49 isolates) of *E. faecium* representing different MTs established high concordance between MLVA and PFGE clusters. However, PFGE was more discriminatory and resolved 8 subtypes (with >92% similarity) within four different MTs (Figure 6).

**Figure 7:**
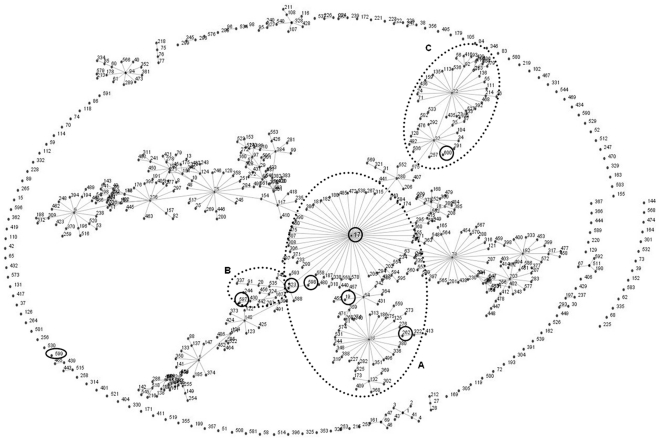
Clustering of eight *E. faecium* STs from the feces of ICU dogs. eBURST clustering of eight *E. faecium* multi-locus sequence types (STs) from this study (indicated by solid line circles) in relation to one representative of 600 STs from MLST database. Each ST is represented as a node, and lines connect single locus variants. Dotted line circles represent STs from A,B: clinical infections, hospital outbreaks; C: hospital and community surveys, environment, and animals (dog, cat, chicken, mouse, ostrich). ICU = intensive care unit.

Furthermore, one representative isolate of each MLVA type was genotyped by MLST. A total of eight different STs were found, out of which four STs were novel (STs 597, 598, 599, and 600). Six STs (17, 323, 597, 598, 19, and 262) were related to isolates from clinical infections or hospital outbreaks with ST-17 as the primary founder (Figure 7). ST-600 placed in the cluster containing primarily isolates from environment, various animals, hospital and community surveys (http://efaecium.mlst.net/). ST-599 was not linked to any other ST in the database. Each ST represented different MTs obtained from this study (ST-17 = MT-1, ST-19 = MT-30, ST-262 = MT-10, ST-323 = MT-12, ST-598 = MT-27, ST-599 = MT-335, and ST-600 = MT-336) except for ST-597 that included MTs 337 and 338 (SLVs to each other).

## Discussion

Multi-drug resistant bacteria in human ICUs and resulting negative impact on treatment outcomes as well as increased treatment costs have been reported in numerous studies [18], [19], [40]–[42]. In contrast, relatively little is known about veterinary ICUs in terms of antibiotic resistant strains, animal nosocomial infections, and potential human health risks [43]. Boerlin *et al.* [44] highlighted the potential problem of nosocomial antibiotic resistance in a veterinary teaching hospital when they examined *Acinetobacter baumanii* and *E. faecium* from infected surgical wounds. Moreover, an increase in the proportion of antibiotic resistant rectal *Escherichia coli* was found associated with longer hospitalization time of dogs [45]. Recently, Clooten *et al.* [46] reported *Clostridium difficile* in 18% of cats and dogs treated (n = 402) in the veterinary ICU. Another recent study provided new information on antibiotic use in critically ill dogs in the small animal ICU in the USA, and reported 19 out of 70 isolates (27.0%) to be multi-drug resistant *A. baumanii*, *E. coli*, and *Enterobacter* spp. [47]. However, the majority of samples (endotracheal washes, urine, and peritoneal fluid) were taken within first 24 h of hospitalization and that may explain a relatively low frequency of multi-drug resistant population. Unfortunately, this study did not focus on the digestive tract microbes and it is therefore not comparable to ours.

In our study, we characterized enterococci isolated from feces of dogs that stayed at the veterinary ICU for 2–9 days and received an antibiotic treatment. In addition, we used the 454 parallel pyrosequencing approach to assess the overall bacterial diversity in the same fecal samples.

### Overall bacterial diversity

Studies assessing canine fecal or digestive tract microbial diversity focused only on healthy animals and it has been reported that irrespective of animal age and breed, major bacterial taxa included *Bacteroidetes*, *Fusobacteria*, lactobacilli and streptococci (*Firmicutes*), whereas the enterococcal, clostridial, bifidobacterial, and eubacterial groups were less prominent [48], [49]. Previously, barcoded pyrosequencing was applied to analyze the fecal microbiota of healthy dogs and showed that the vast majority (>99%) belonged to the five phyla that we also detected in our ICU dogs; however, phyla *Sprirochetes* and *Tenericutes* were found only in healthy animals [23]. Another pyrosequencing based study reported *γ*-*Proteobacteria* as the most dominant group in the small intestine of dogs at pre- and post-tylosin treatment [50]. In contrast, the abundance of *γ*-*Proteobacteria* in our study was low (<16.5% of all sequences) with the exception of the dog ICU-7 where it comprised 47.7%. Members of the phyla *Firmicutes* and *Actinobacteria* are well-known for their ability to withstand harsh conditions and for their resistance to various antibiotics [51], [52]. Here we report remarkably high proportion of *Firmicutes* in 5 out of 7 dogs (76.0–98.9%) compared to published data on healthy dogs (in feces: 14–28%, [23]; in the digestive tract: ∼40% [51]). This is likely result of changes in the gut microbiome due to animal sickness, stress, and antibiotic treatment. However, the actinobacterial population in the ICU dogs (1.6±1.0%) was comparable to that of healthy dogs (0.8–1.4%) [23]. The phylum *Fusobacteria* was represented by the sole genus *Fusobacterium* in both ICU and healthy dogs. It is important to keep in mind however, that the PCR and pyrosequencing approach assessing diversity of complex microbial communities may introduce some bias towards specific bacterial taxa due to differences in cell lyses and primer annealing.

The pyrosequencing-based datasets with relatively high number of reads (range: 2,600–9,300 with a mean of ∼4,000 reads per sample) were unable to fully cover the diversity of the fecal microbiota of healthy dogs [23]. Despite a relatively low number of sequences, our dataset (∼2,300 reads per sample) indicates the low microbial diversity and dominance of one to two genera in the entire community in six out of seven dogs. The most striking observation obtained from pyrosequencing data was on the genus level where enterococci made an abnormally high proportion of the fecal microbiota in 5 out of 7 dogs. Previous culture-dependent as well as culture-independent studies showed that enterococci make up less than 1.0% of the intestinal microbiota of healthy dogs [53] which is typical for the digestive tract microbiota of other mammals including people [54]. The mean of the enterococcal community size (48.9±11.5%) in the ICU dogs was 50–100 fold higher when compared to that of the healthy dogs intestinal microbiota (0.1–0.5%) [51] or even dogs treated with antibiotic tylosin (1.1%) [52]. Our pyrosequencing results were further supported by the culture-dependent technique where the enterococcal concentration (1.4±0.8×10^8^ g^−1^ feces) in the ICU dogs was up to 100 fold higher than that (10^3^–10^6^ CFU g^−1^ feces) reported from the healthy canine gut [15]. To our knowledge, this is the first study reporting such a high enterococcal population in the digestive tract of diseased dogs irrespective of the class of antibiotic used. Overall, antibiotic treatments and animal health condition did not disturb the composition of the microbiota at the phylum level; however, there was a great variation in the microbial community among dogs on the genus level.

The resolution of our dataset based on 16S rDNA decreased beyond the genus level depending on the length of good-quality sequence and on the specific bacterial taxon, yet a glimpse of species richness was obtained. It is worrisome to note that *Streptococcus gallolyticus*, *Salmonella enterica*, *Shigella boydii* and several strains of *Clostridium* (*C. difficile*, *C. symbiosum*, *C. glycolicum*, *C. baratii*, *C. sordellii*, *C. perfringens*) that can have a negative effect on human as well as animal health [55], were tentatively detected in some of the ICU dogs though at a relatively low frequency. Therefore, the risk associated with ill dogs is not limited to shedding high numbers of antibiotic resistant enterococci as the digestive tract of these animals may contain other bacteria of public health importance.

The number of OTUs in the feces of our ICU dogs was about three fold lower than that in the digestive tract [51] and feces [23] of healthy dogs. Shannon diversity index (H′) in healthy dogs was almost double of that in the ICU dogs [23], [51]. Moreover, compared to our results, the species richness (OTUs = 149) and diversity (H′ = 2.84) as well as ACE (209) and Chao1 (204) estimates were much higher in the study on the canine jejunum microbiota after tylosin treatment [52]. Nevertheless, rarefaction values did not reach a plateau and thereby additional sequencing to assess the diversity in greater depth is warranted.

Culture-based as well as culture-independent studies of the healthy canine feces revealed a diverse enterococcal community that typically included *E. faecalis*, *E. faecium*, *E. hirae*, *E. avium*, *E. raffinosus*, *E. durans*, *E. casseliflavus*, *E. gallinarum*, *E. mundtii*, and *E. canintestini* [16], [56]–[59]. In contrast, feces of the ICU dogs in our study contained only two species, *E. faecalis* and *E. faecium*, and three out of 7 dogs had exclusively only one of these. Such reduced diversity further likely reflects the effect of antibiotic therapy on the enterococcal population.

### Antibiotic resistance of enterococci

A number of studies in different parts of Europe demonstrated reduced susceptibility to several antibiotics including tetracycline, erythromycin, ampicillin, enrofloxacin, ciprofloxacin, and rifampicin among *E. faecalis* and *E. faecium* isolated from healthy pets (dogs and cats) [14], [60]–[62]. Furthermore, enterococcal isolates from diseased dogs under *β*-lactam treatment in the UK and Denmark showed high resistance to ampicillin (100%), erythromycin (97%), ciprofloxacin (92%), tetracycline (89%), rifampin (54%), followed by low frequency of resistance to gentamicin (5%), linezolid (3%), and streptogramins (2%) [15]. In a veterinary teaching hospital in Canada, a significant increase in the proportion of multi-drug resistant enterococcal urinary tract infections (UTI) in dogs over a 15 year period was documented [63]. The only study conducted in the United States included on an average 1–2 enterococcal isolates from each of 155 dogs and 121 cats representing an overview of the prevalence of antibiotic resistance in the healthy small animal population [16]. Consequently, the species diversity and actual shedding of resistant enterococci from individual animals could not be evaluated. Though multi-drug resistant *E. faecalis* and *E. faecium* were detected in healthy pets, no supportive information was available on the history of their antibiotic exposure [16]. In our study, a strong association was found between the antibiotic treatment and resistant population in two instances where enterococcal isolates from the dog ICU-1 treated with doxycycline and the dog ICU-5 treated with ampicillin showed 90% and 100% resistance to the respective antibiotics. The dog ICU-6 received cefpodoxime treatment and since enterococci are intrinsically resistant to 3^rd^ generation cephalosporins, they were not affected directly. However, cefpodoxime probably greatly affected other bacteria in the digestive tract of this dog resulting in the major shift towards enterococci. Fortunately, unlike in Europe [64]–[66], all fecal isolates from the ICU dogs were susceptible to vancomycin as well as drugs for the second-line of treatment including linezolid, quinupristin/dalfopristin, and tigecycline likely due of their restricted and judicious use in the USA.

### Horizontal transfer of resistance traits of *E. faecium*

In the last two decades, multi-drug resistant *E. faecium* has emerged as a serious nosocomial pathogen in human hospitals [67]. Ike *et al.* [27] described intra- as well as inter-species transfer of the gentamicin resistance trait from *E. faecium* via a pheromone-independent conjugal plasmid pMG1 with a transfer frequency of 10^−4^ per donor cell in broth mating, which is similar to the intra-species transfer rate (10^−3^ per donor) in the ICU dog isolates in both, broth and filter mating, assays. Transfer frequency (10^−7^–10^−5^) for erythromycin resistance among *E. faecium* in both mating assays also fell within the range that is typically mediated by conjugative transposons [68]. In a recent study by Arias *et al.* [69], transfer of genes for streptomycin resistance has been demonstrated with a rate of 10^−5^ per donor cell in filter mating, which is higher than that (10^−7^) determined in our study. The low transfer rate indicated that this resistance trait may not be carried by high frequency transferable plasmids carrying other virulence factors such as *hyl_Efm_* [69]. Intra-species transfer (10^−5^ in broth mating and 10^−4^ in filter mating) of tetracycline resistance in enterococci has been shown involving conjugative transposon Tn*916* [70], whereas transfer of resistance to doxycycline has only been observed in *Streptococcus sanguinus* via conjugative transposon CTn*6002* (a complex element partly derived from Tn*916*) [71]. Our data revealed a high transfer rate (10^−4^–10^−5^) for both, tetracycline and doxycycline resistance traits, indicating potential involvement of Tn*916*. Ampicillin and enrofloxacin resistance traits could not be transferred *in vitro*, possibly because the conferring resistance genes such as *pbp5* or *gyrA* are commonly encoded on non-transferable regions of the chromosome [72], [73].

This part of the study demonstrated that companion animals after release from the ICU and on antibiotic treatment harbor a large multi-drug resistant enterococcal community. Consequently, the spread of antibiotic resistant strains and resistance traits via the companion animals is plausible. Transmission of pathogenic strains of methicillin resistant staphylococci [5], [74] and vancomycin resistant enterococci [7], [75] between domestic/clinic pets and people (owners/clinic staff) has been suggested previously.

### Virulence traits and biofilm formation

*E. faecalis* strains are well-known for their association with endocarditis, bacteremia, and UTIs due, in part, to virulence factors such as GelE and Esp [76]. Presence of *gelE* and *esp* has been reported to enhance biofilm formation by *E. faecalis* and *E. faecium* and this likely confers a significant survival advantage by increasing bacterial resistance to stressful environmental conditions and antimicrobial exposure [35], [36], [77]–[79]. It is noteworthy that most of the *E. faecalis* from the ICU dogs harbored and expressed either *gelE* or *esp* or both and formed biofilm *in vitro*, suggesting that these isolates may be opportunistically pathogenic under suitable conditions. Aggregation substance (AS) along with enterococcal binding substance (EBS) has been shown to be associated with *E. faecalis* mediated endocarditis [80]. Despite the presence of *asa1* (one of the genes encoding AS) in a large portion of *E. faecalis* from the ICU dogs, these strains were phenotypically negative *in vitro*. It is likely that specific *in vivo* conditions are required for the expression of *asa1* [81]. Cytolysin is a unique secreted bacterial toxin hemolytic to human, horse, and rabbit erythrocytes and it also is bactericidal to other Gram-positive bacteria [82]. Presence of *cylA* (one of the genes encoding cytolysin) in *E. faecalis* and corresponding *β*-hemolytic activity may not only make these strains more pathogenic, but also might facilitate the competition with other Gram-positive bacteria in the gut. Since the gut of ICU dogs was overpopulated with enterococci, there was a high likelihood of triggering quorum sensing that could activate *gelE* as well as *cylA in vivo* [83], [84].

In contrast, while many *E. faecium* also tested positive for *gelE* and *cylA*, none of them were positive for the strong gelatinase phenotype and *β-*hemolysis, respectively. Although few other studies [85], [86] reported presence of silent *gelE* and *cylA* in *E. faecium*, both of these genes are not common in this species [33]. Overall, *E. faecalis* expressed more virulence traits than *E. faecium* possibly reflecting its greater prevalence in enterococcal nosocomial infections [11].

### Genotypic diversity

Genetic similarities between multi-drug resistant enterococcal strains isolated from dogs and humans/hospitalized patients have been evident from fingerprinting techniques such as AFLP [87], PFGE [75], and MLST [14], [15]. MLVA has also been successfully introduced for genotyping *E. faecium* from large nosocomial outbreaks [88]. MLVA clustering analysis of multi-drug resistant *E. faecium* from the ICU dogs portrayed their lineages and the global epidemiology. Interestingly, only one MT from the ICU dogs clustered with the clones obtained from dogs previously while seven out of 9 MTs were related to MTs from human clinical infections and hospital outbreaks. MLVA data supported by PFGE analysis indicated a low genotypic diversity of *E. faecium* likely reflecting the antibiotic selective pressure. Although the majority of our strains were host specific (unique MTs and pulsotypes in individual dogs); interestingly, sharing of three *E. faecium* clones among four dogs suggested a possible nosocomial origin of these strains. In contrast, high genotypic diversity of *E. faecium* was detected in healthy dogs without antibiotic selective pressure [17].

Based on MLST analysis, the *E. faecium* disseminated in human hospitals in several parts of the world belongs to the clonal complex 17 (CC-17) containing several sub-complexes [67], [89], [90]. Cluster analysis based on MLST further confirmed close relation of *E. faecium* from the ICU dogs in our study and strains from human clinical infections and hospital outbreaks. One ST belonged to the CC-17 (ST-17) and four other clonal types were directly or indirectly (via sub-complex ST-18) linked to CC-17 as well. Only one of the clones (a new ST-600) from dog ICU-6 clustered with other isolates from pets. Number of genotypes generated by MLST and MLVA were in good agreement with one exception where two different MTs were found to be identical based on MLST analysis. It has been established that the gradual accrual of virulence factors (*esp*, *fms*, *hyl*) and resistance genes (for streptomycin, ampicillin, gentamicin, and vancomycin) resulted in the formation of the genogroup CC-17 [91], [92]. Damborg *et al.* [15] demonstrated that canine *E. faecium* isolates, in spite of the lack of *esp* and *hyl*, were related to hospital associated *E. faecium* clones (ST-78 and ST-192). It is interesting to note that *E. faecium* from our ICU dogs, though also negative for *esp*, were resistant to ampicillin and high concentration aminoglycosides and this further emphasizes their potential connection to human clinical clones.

In summary, the dogs after release from the ICU and on an antibiotic treatment harbored a very large multi-drug resistant population of *E. faecalis* and/or *E. faecium*. The ability to transfer the resistance traits horizontally, presence of virulence factors as well as biofilm forming capacity underline the potential clinical importance of these strains. The diversity of the overall fecal microbiota of the treated dogs was low. In addition, genotyping of *E. faecium* strains revealed very low clonal diversity, their possible nosocomial origin, and close relatedness to human clinical isolates. While the temporal effect of antibiotic treatment on the canine gut microbial community and its antibiotic resistance profile remains to be determined, based on results of several studies with human and mouse microbiota [93]–[95], it may be long lasting. Prudent use of antibiotics in veterinary medicine is critical in order to avoid treatment failures and zoonotic spread of multi-drug resistant bacterial strains. Importantly, restricted contact between treated dogs and their owners is recommended to avoid health risks.

## Supporting Information

Table S1 Information on dogs from the intensive care unit (ICU).
(DOC)

Table S2 Number of operational taxonomic units estimated in the feces of seven dogs from the intensive care unit (ICU) and corresponding diversity indices and coverage percentages.
(DOC)

Table S3 Distribution (%) of identified sequences from the feces of seven dogs from the intensive care unit (ICU) based on 454 pyrosequencing data of 16S rDNA.
(DOC)

Table S4 Multiple (≥3) antibiotic resistance profile among enterococci from the feces of dogs from the intensive care unit (ICU).
(DOC)

Table S5 (A) Antibiotic resistance phenotype and virulence genotypic profile of *E. faecalis* from individual ICU dogs. (B) Antibiotic resistance phenotype, virulence genotypic profile, and MLVA types (MTs) of *E. faecium* from individual ICU dogs. Isolates are grouped (color coded) based on their antibiogram. ICU = intensive care unit, R = resistance to antibiotics, ‘+’ = presence of virulence gene, NT = not typeable.
(PDF)

## References

[1] Herrero I, Fernández-Garayzábal JF, Moreno MA, Domínguez L (2004). Dogs should be included in surveillance programs for vancomycin-resistant enterococci.. J Clin Microbiol.

[2] Guardabassi L, Schwarz S, Lloyd DH (2004a). Pet animals as reservoirs of antimicrobial-resistant bacteria.. J Antimicrob Chemother.

[3] Manian FA (2003). Asymptomatic nasal carriage of mupirocin-resistant, methicillin-resistant *Staphylococcus aureus* (MRSA) in a pet dog associated with MRSA infection in household contacts.. Clin Infect Dis.

[4] Guardabassi L, Loeber ME, Jacobson A (2004b). Transmission of multiple antimicrobial-resistant *Staphylococcus intermedius* between dogs affected by deep pyoderma and their owners.. Vet Microbiol.

[5] Frank LA, Kania SA, Kirzeder EM, Eberlein LC, Bemis DA (2009). Risk of colonization or gene transfer to owners of dogs with methicillin-resistant *Staphylococcus pseudintermedius*.. Vet Dermatol.

[6] Wolfs TF, Duim B, Geelen SP, Rigter A, Thomson-Carter F (2001). Neonatal sepsis by *Campylobacter jejuni*: genetically proven transmission from a household puppy.. Clin Infect Dis.

[7] Simjee S, White DG, McDermott PF, Wagner DD, Zervos MJ (2002). Characterization of Tn*1546* in vancomycin-resistant *Enterococcus faecium* isolated from canine urinary tract infections: evidence of gene exchange between human and animal enterococci.. J Clin Microbiol.

[8] Bell JA, Kopper JJ, Turnbull JA, Barbu NI, Murphy AJ (2008). Ecological characterization of the colonic microbiota of normal and diarrheic dogs.. Interdiscip Perspect Infect Dis.

[9] Murray BE (2000). Vancomycin-resistant enterococcal infections.. N Engl J Med.

[10] Arias CA, Murray BE (2008). Emergence and management of drug-resistant enterococcal infections.. Expert Rev Anti Infect Ther.

[11] Hidron AI, Edwards JR, Patel J, Horan TC, Sievert DM (2008). NHSN annual update: antimicrobial-resistant pathogens associated with healthcare-associated infections: annual summary of data reported to the National Healthcare Safety Network at the Centers for Disease Control and Prevention, 2006–2007.. Infect Control Hosp Epidemiol.

[12] Dzidic S, Bedeković V (2003). Horizontal gene transfer-emerging multidrug resistance in hospital bacteria.. Acta Pharmacol Sin.

[13] Coburn PS, Baghdayan AS, Dolan GT, Shankar N (2007). Horizontal transfer of virulence genes encoded on the *Enterococcus faecalis* pathogenicity island.. Mol Microbiol.

[14] Damborg P, Sørensen AH, Guardabassi L (2008). Monitoring of antimicrobial resistance in healthy dogs: first report of canine ampicillin-resistant *Enterococcus faecium* clonal complex 17.. Vet Microbiol.

[15] Damborg P, Top J, Hendrickx APA, Dawson S, Willems RJL (2009). Dogs are a reservoir of ampicillin-resistant *Enterococcus faecium* lineages associated with human infections.. Appl Environ Microbiol.

[16] Jackson CR, Fedorka-Cray PJ, Davis JA, Barrett JB, Frye JG (2009a). Prevalence, species distribution and antimicrobial resistance of enterococci isolated from dogs and cats in the United States.. J Appl Microbiol.

[17] Jackson CR, Fedorka-Cray PJ, Davis JA, Barrett JB, Brousse JH (2009b). Mechanisms of antimicrobial resistance and genetic relatedness among enterococci isolated from dogs and cats in the United States.. J Appl Microbiol.

[18] Austin DJ, Bonten MJM, Weinstein RA, Slaughter S, Anderson RM (1999). Vancomycin-resistant enterococci in intensive-care hospital settings: transmission dynamics, persistence, and the impact of infection control programs.. Proc Natl Acad Sci USA.

[19] Samuelsson A, Jonasson J, Monstein HJ, Berg S, Isaksson B (2003). Clustering of enterococcal infections in a general intensive care unit.. J Hosp Infect.

[20] Kramer A, Schwebke I, Kampf G (2006). How long do nosocomial pathogens persist on inanimate surfaces? A systematic review.. BMC Infect Dis.

[21] Macovei L, Zurek L (2007). Influx of enterococci and associated antibiotic resistance and virulence genes from ready-to-eat food to the human digestive tract.. Appl Environ Microbiol.

[22] Dowd SE, Callaway TR, Wolcott RD, Sun Y, McKeehan T (2008). Evaluation of the bacterial diversity in the feces of cattle using 16S rDNA bacterial tag-encoded FLX amplicon pyrosequencing (bTEFAP).. BMC Microbiol.

[23] Middelbos IS, Vester Boler BM, Qu A, White BA, Swanson KS (2010). Phylogenetic characterization of fecal microbial communities of dogs fed diets with or without supplemental dietary fiber using 454 pyrosequencing.. PLoS ONE.

[24] Schloss PD, Westcott SL, Ryabin T, Hall JR, Hartmann M (2009). Introducing mothur: open-source, platform-independent, community-supported software for describing and comparing microbial communities.. Appl Environ Microbiol.

[25] Clinical and Laboratory Standards Institute (2008). Performance standards for antimicrobial disk and dilution susceptibility tests for bacteria isolated from animals. Approved Standard CLSI Document M31-A3, 3rd edition.

[26] Clinical and Laboratory Standards Institute (2010). Performance standards for antimicrobial susceptibility testing; twentieth informational supplement. CLSI Document M100-S20.

[27] Ike Y, Tanimoto K, Tomita H, Takeuchi K, Fujimoto S (1998). Efficient transfer of the pheromone-independent *Enterococcus faecium* plasmid pMG1 (Gm^r^) (65.1 kilobases) to *Enterococcus* strains during broth mating.. J Bacteriol.

[28] Tendolkar PM, Baghdayan AS, Shankar N (2006). Putative surface proteins encoded within a novel transferable locus confer a high-biofilm phenotype to *Enterococcus faecalis*.. J Bacteriol.

[29] Nannini EC, Pai SR, Singh KV, Murray BE (2003). Activity of tigecycline (GAR-936), a novel glycylcycline, against enterococci in the mouse peritonitis model.. Antimicrob Agents Chemother.

[30] Coque TM, Tomayko JF, Ricke SC, Okhyusen PC, Murray BE (1996). Vancomycin-resistant enterococci from nosocomial, community, and animal sources in the United States.. Antimicrob Agents Chemother.

[31] Landman D, Mobarakai NK, Quale JM (1993). Novel antibiotic regimens against *Enterococcus faecium* resistant to ampicillin, vancomycin, and gentamicin.. Antimicrob Agents Chemother.

[32] Qi C, Zheng X, Obias A, Scheetz MH, Malczynski M (2006). Comparison of testing methods for detection of decreased linezolid susceptibility due to G2576T mutation of the 23S rRNA gene in *Enterococcus faecium* and *Enterococcus faecalis*.. J Clin Microbiol.

[33] Vankerckhoven V, Autgaerden TV, Vael C, Lammens C, Chapelle S (2004). Development of a multiplex PCR for the detection of *asa1*, *gelE*, *cylA*, *esp*, and *hyl* genes in enterococci and survey for virulence determinants among European hospital isolates of *Enterococcus faecium*.. J Clin Microbiol.

[34] Macovei L, Zurek L (2006). Ecology of antibiotic resistance genes: characterization of enterococci from houseflies collected in food settings.. Appl Environ Microbiol.

[35] Macovei L, Ghosh A, Thomas VC, Hancock LE, Mahmood S (2009). *Enterococcus faecalis* with the gelatinase phenotype regulated by the *fsr* operon and with biofilm-forming capacity are common in the agricultural environment.. Environ Microbiol.

[36] Hancock LE, Perego M (2004). The *Enterococcus faecalis fsr* two-component system controls biofilm development through production of gelatinase.. J Bacteriol.

[37] Top J, Schouls LM, Bonten MJM, Willems RJ (2004). Multiple-locus variable-number tandem repeat analysis, a novel typing scheme to study the genetic relatedness and epidemiology of *Enterococcus faecium* isolates.. J Clin Microbiol.

[38] Feil EJ, Li BC, Aanensen DM, Hanage WP, Spratt BG (2004). eBURST: inferring patterns of evolutionary descent among clusters of related bacterial genotypes from multilocus sequence typing data.. J Bacteriol.

[39] Amachawadi RG, Shelton NW, Jacob ME, Shi X, Narayanan SK (2010). Occurrence of *tcrb*, a transferable copper resistance gene, in fecal enterococci of swine.. Foodborne Pathog Dis.

[40] Pelz RK, Lipsett PA, Swoboda SM, Diener-West M, Powe NR (2002). Vancomycin-sensitive and vancomycin-resistant enterococcal infections in the ICU: attributable costs and outcomes.. Intensive Care Med.

[41] Roberts RR, Hota B, Ahmad I, Scott RD, Foster SD (2009). Hospital and societal costs of antimicrobial-resistant infections in a Chicago teaching hospital: implications for antibiotic stewardship.. Clin Infect Dis.

[42] Wassenberg MWM, de Wit GA, van Hout BA, Bonten MJM (2010). Quantifying cost-effectiveness of controlling nosocomial spread of antibiotic-resistant bacteria: the case of MRSA.. PLoS ONE.

[43] Umber JK, Bender JB (2009). Pets and antimicrobial resistance.. Vet Clin North Am Small Anim Pract.

[44] Boerlin P, Eugster S, Gaschen F, Straub R, Schawalder P (2001). Transmission of opportunistic pathogens in a veterinary teaching hospital.. Vet Microbiol.

[45] Ogeer-Gyles JS, Mathews KA, Boerlin P (2006). Nosocomial infections and antimicrobial resistance in critical care medicine.. J Vet Emerg Crit Care.

[46] Clooten J, Kruth S, Arroyo L, Weese JS (2008). Prevalence and risk factors for *Clostridium difficile* colonization in dogs and cats hospitalized in an intensive care unit.. Vet Microbiol.

[47] Black DM, Rankin SC, King LG (2009). Antimicrobial therapy and aerobic bacteriologic culture patterns in canine intensive care unit patients: 74 dogs (January–June 2006).. J Vet Emerg Crit Care.

[48] Simpson JM, Martineau B, Jones WE, Ballam JM, Mackie RI (2002). Characterization of fecal bacterial populations in canines: effects of age, breed and dietary fiber.. Microb Ecol.

[49] Suchodolski JS, Camacho J, Steiner JM (2008). Analysis of bacterial diversity in the canine duodenum, jejunum, ileum, and colon by comparative 16S rRNA gene analysis.. FEMS Microbiol Ecol.

[50] Suchodolski JS, Dowd SE, Westermarck E, Steiner JM, Wolcott RD (2009). The effect of the macrolide antibiotic tylosin on microbial diversity in the canine small intestine as demonstrated by massive parallel 16S rRNA gene sequencing.. BMC Microbiol.

[51] Teixeira LCRS, Peixoto RS, Cury JC, Sul WJ, Pellizari VH (2010). Bacterial diversity in rhizosphere soil from Antarctic vascular plants of Admiralty Bay, maritime Antarctica.. ISME J.

[52] Ventura M, Canchaya C, Tauch A, Chandra G, Fitzgerald GF (2007). Genomics of *Actinobacteria*: tracing the evolutionary history of an ancient phylum.. Microbiol Mol Biol Rev.

[53] Mentula S, Harmoinen J, Heikkila M, Westermarck E, Rautio M (2005). Comparison between cultured small-intestinal and fecal microbiotas in Beagle dogs.. Appl Environ Microbiol.

[54] Sghir A, Gramet G, Suau A, Rochet V, Pochart P (2000). Quantification of bacterial groups within human fecal flora by oligonucleotide probe hybridization.. Appl Environ Microbiol.

[55] Scallan E, Hoekstra RM, Angulo FJ, Tauxe RV, Widdowson MA (2011). Foodborne illness acquired in the United States - major pathogens.. Emerg Infect Dis.

[56] Devriese LA, Van De Kerckhove A, Kilpper-Bälz R, Schleifer KH (1987). Characterization and identification of *Enterococcus* species isolated from the intestines of animals.. Int J Syst Bacteriol.

[57] Devriese LA, Colque JIC, De Herdt P, Haesebrouck F (1992). Identification and composition of the tonsillar and anal enterococcal and streptococcal flora of dogs and cats.. J Appl Microbiol.

[58] Graef EM, Devriese LA, Baele M, Vancanneyt M, Swings J (2005). Identification of enterococcal, streptococcal and *Weissella* species in the faecal flora of individually owned dogs.. J Appl Microbiol.

[59] Kim SY, Adachi Y (2007). Biological and genetic classification of canine intestinal lactic acid bacteria and bifidobacteria.. Microbiol Immunol.

[60] Butaye P, Devriese LA, Haesebrouck F (2001). Differences in antibiotic resistance patterns of *Enterococcus faecalis* and *Enterococcus faecium* strains isolated from farm and pet animals.. Antimicrob Agents Chemother.

[61] Poeta P, Costa D, Rodrigues J, Torres C (2006). Antimicrobial resistance and the mechanisms implicated in faecal enterococci from healthy humans, poultry and pets in Portugal.. Int J Antimicrob Agents.

[62] Ossiprandi MC, Bottarelli E, Cattabiani F, Bianchi E (2008). Susceptibility to vancomycin and other antibiotics of 165 *Enterococcus* strains isolated from dogs in Italy.. Comp Immunol Microbiol Infect Dis.

[63] Prescott JF, Brad Hanna WJ, Reid-Smith R, Drost K (2002). Antimicrobial drug use and resistance in dogs.. Can Vet J.

[64] van Belkum A, van den Braak N, Thomassen R, Verbrugh H, Endtz H (1996). Vancomycin-resistant enterococci in cats and dogs.. Lancet.

[65] Devriese LA, Ieven M, Goossens H, Vandamme P, Pot B (1996). Presence of vancomycin-resistant enterococci in farm and pet animals.. Antimicrob Agents Chemother.

[66] De Leener E, Decostere A, De Graef EM, Moyaert H, Haesebrouck F (2005). Presence and mechanism of antimicrobial resistance among enterococci from cats and dogs.. Microb Drug Resist.

[67] Willems RJ, van Schaik W (2009). Transition of *Enterococcus faecium* from commensal organism to nosocomial pathogen: natural ecology of *Enterococcus faecium*.. Future Microbiol.

[68] Takeuchi K, Tomita H, Fujimoto S, Kudo M, Kuwano H (2005). Drug resistance of *Enterococcus faecium* clinical isolates and the conjugative transfer of gentamicin and erythromycin resistance traits.. FEMS Microbiol Lett.

[69] Arias CA, Panesso D, Singh KV, Rice LB, Murray BE (2009). Cotransfer of antibiotic resistance genes and a *hyl_efm_*-containing virulence plasmid in *Enterococcus faecium*.. Antimicrob Agents Chemother.

[70] Bentorcha F, de Cespedes G, Horaud T (1991). Tetracycline resistance heterogeneity in *Enterococcus faecium*.. Antimicrob Agents Chemother.

[71] Warburton PJ, Palmer RM, Munson MA, Wade WG (2007). Demonstration of *in vivo* transfer of doxycycline resistance mediated by a novel transposon.. J Antimicrob Chemother.

[72] Rice LB, Carias LL, Hutton-Thomas R, Sifaoui F, Gutmann L (2001). Penicillin-binding protein 5 and expression of ampicillin resistance in *Enterococcus faecium*.. Antimicrob Agents Chemother.

[73] el Amin NA, Jalal S, Wretlind B (1999). Alterations in GyrA and ParC associated with fluoroquinolone resistance in *Enterococcus faecium*.. Antimicrob Agents Chemother.

[74] Loeffler A, Boag AK, Sung J, Lindsay JA, Guardabassi L (2005). Prevalence of methicillin-resistant *Staphylococcus aureus* among staff and pets in a small animal referral hospital in the UK.. J Antimicrob Chemother.

[75] Manson JM, Keis S, Smith JMB, Cook GM (2003). Characterization of a vancomycin-resistant *Enterococcus faecalis* (VREF) isolate from a dog with mastitis: further evidence of a clonal lineage of VREF in New Zealand.. J Clin Microbiol.

[76] Gilmore MS, Coburn PS, Nallapareddy SR, Murray BE, Gilmore MS, Clewell DB, Courvalin P, Dunny GM, Murray BE (2002). Enterococcal virulence.. The enterococci: pathogenesis, molecular biology and antibiotic resistance.

[77] Sandoe JAT, Witherden IR, Cove JH, Heritage J, Wilcox MH (2003). Correlation between enterococcal biofilm formation *in vitro* and medical-device-related infection potential *in vivo*.. J Med Microbiol.

[78] Tendolkar PM, Baghdayan AS, Gilmore MS, Shankar N (2004). Enterococcal surface protein, Esp, enhances biofilm formation by *Enterococcus faecalis*.. Infect Immun.

[79] Heikens E, Bonten MJM, Willems RJL (2007). Enterococcal surface protein Esp is important for biofilm formation of *Enterococcus faecium* E1162.. J Bacteriol.

[80] Schlievert PM, Gahr PJ, Assimacopoulos AP, Dinges MM, Stoehr JA (1998). Aggregation and binding substances enhance pathogenicity in rabbit models of *Enterococcus faecalis* endocarditis.. Infect Immun.

[81] Chow JW, Thal LA, Perri MB, Vazquez JA, Donabedian SM (1993). Plasmid-encoded hemolysin and aggregation substance production contribute to virulence in experimental enterococcal endocarditis.. Antimicrob Agents Chemother.

[82] Shankar N, Coburn P, Pillar C, Haas W, Gilmore M (2004). Enterococcal cytolysin: activities and association with other virulence traits in a pathogenicity island.. Int J Med Microbiol.

[83] Nakayama J, Cao Y, Horii T, Sakuda S, Akkermans ADL (2001). Gelatinase biosynthesis-activating pheromone, a peptide lactone that mediates a quorum sensing in *Enterococcus faecalis*.. Mol Microbiol.

[84] Haas W, Shepard BD, Gilmore MS (2002). Two-component regulator of *Enterococcus faecalis* cytolysin responds to quorum-sensing autoinduction.. Nature.

[85] Eaton TJ, Gasson MJ (2001). Molecular screening of *Enterococcus* virulence determinants and potential for genetic exchange between food and medical isolates.. Appl Environ Microbiol.

[86] Biavasco F, Foglia G, Paoletti C, Zandri G, Magi G (2007). VanA-type enterococci from humans, animals, and food: species distribution, population structure, Tn*1546* typing and location, and virulence determinants.. Appl Environ Microbiol.

[87] Willems RJ, Top J, van den Braak N, van Belkum A, Endtz H (2000). Host specificity of vancomycin-resistant *Enterococcus faecium*.. J Infect Dis.

[88] Valdezate S, Labayru C, Navarro A, Mantecón MA, Ortega M (2009). Large clonal outbreak of multidrug-resistant CC17 ST17 *Enterococcus faecium* containing Tn*5382* in a Spanish hospital.. J Antimicrob Chemother.

[89] Willems RJ, Top J, van Santen M, Robinson DA, Coque TM (2005). Global spread of vancomycin-resistant *Enterococcus faecium* from distinct nosocomial genetic complex.. Emerg Infect Dis.

[90] Freitas AR, Novais C, Ruiz-Garbajosa P, Coque TM, Peixe L (2009). Dispersion of multidrug-resistant *Enterococcus faecium* isolates belonging to major clonal complexes in different Portuguese settings.. Appl Environ Microbiol.

[91] Top J, Willems RJ, Bonten MJM (2008). Emergence of CC17 *Enterococcus faecium*: from commensal to hospital-adapted pathogen.. FEMS Immunol Med Microbiol.

[92] Galloway-Pena JR, Nallapareddy SR, Arias CA, Eliopoulos GM, Murray BE (2009). Analysis of clonality and antibiotic resistance among early clinical isolates of *Enterococcus faecium* in the united states.. J Infect Dis.

[93] Dethlefsen L, Huse S, Sogin ML, Relman DA (2008). The pervasive effects of an antibiotic on the human gut microbiota, as revealed by deep 16S rRNA sequencing.. PLoS Biol.

[94] Antonopoulos DA, Huse SM, Morrison HG, Schmidt TM, Sogin ML (2009). Reproducible community dynamics of the gastrointestinal microbiota following antibiotic perturbation.. Infect Immun.

[95] Jernberg C, Löfmark S, Edlund C, Jansson JK (2010). Long-term impacts of antibiotic exposure on the human intestinal microbiota.. Microbiology.

