# Supplementary material for: Dogs Leaving the ICU Carry a Very Large Multi-Drug Resistant Enterococcal Population with Capacity for Biofilm Formation and Horizontal Gene Transfer
Source: PLoS One. 2011 Jul 19;6(7):e22451. doi: 10.1371/journal.pone.0022451 (PMC3139645; doi:10.1371/journal.pone.0022451)
Supplement: Table S1 — Information on dogs from the intensive care unit (ICU). (DOC) [file pone.0022451.s001.doc]

**Table S1.** Information on dogs from the intensive care unit (ICU).

| **Sample ID.** | **Age/Sex/ Breed** | **Stay at ICU (days)** | **Disease(s) treated** | **Antibiotic(s) used** | **Disease history** |
| --- | --- | --- | --- | --- | --- |
| ICU-1 | 2 yr/ FS/ labradoodle | 2 | immune-mediated anemia | doxycycline | immune-mediated hemolytic anemia |
| ICU-2 | 14 yr/ FS/ Australian blue heeler mix | 2 | urinary tract infection, high fever | clavamox, ampicillin, enrofloxacin | urinary tract infection |
| ICU-3 | 2 yr/ FS/ Yorkshire terrier | 9 | pneumonia | amoxicillin, enrofloxacin | portosystemic shunt |
| ICU-4 | 9 yr/ MC/ labrador | 7 | acute liver failure, fever of unknown origin | ampicillin IV, enrofloxacin | epilepsy and liver disease |
| ICU-5 | 2 months/ MI/ dachshund | 3 | pneumonia | ampicillin IV, clavamox | pneumonia |
| ICU-6 | 4 yr/ MC/  corgi | 2 | pyoderma | cefpodoxime | pyoderma (skin infections) |
| ICU-7 | 1 yr/ FS/ mini schnauzer | 6 | liver disease/ portosystemic shunt | ampicillin | portosystemic shunt |

**Abbr.** FS, Female spayed; MC, male castrate; MI, male intact.
