# Supplementary material for: Dogs Leaving the ICU Carry a Very Large Multi-Drug Resistant Enterococcal Population with Capacity for Biofilm Formation and Horizontal Gene Transfer
Source: PLoS One. 2011 Jul 19;6(7):e22451. doi: 10.1371/journal.pone.0022451 (PMC3139645; doi:10.1371/journal.pone.0022451)
Supplement: Table S2 — Number of operational taxonomic units estimated in the feces of seven dogs from the intensive care unit (ICU) and corresponding diversity indices and coverage percentages. (DOC) [file pone.0022451.s002.doc]

**Table S2.** Number of operational taxonomic units estimated in feces of seven dogs from the intensive care unit (ICU) and corresponding diversity indices and coverage percentages.

| **Distance*** | | **0.01** | | | | | **0.03** | | | | | **0.05** | | | | |
| --- | --- | --- | --- | --- | --- | --- | --- | --- | --- | --- | --- | --- | --- | --- | --- | --- |
| **Sample ID.** | **Reads** | **Rf** | **OTU** | **H’** | **ACE** | **Chao1** | **Rf** | **OTU** | **H’** | **ACE** | **Chao1** | **Rf** | **OTU** | **H’** | **ACE** | **Chao1** |
| ICU-1 | 2201 | 162.9 | 170 | 2.6 | 225.2 | 218.7 | 85.9 | 89 | 1.6 | 93.9 | 78.5 | 62.5 | 64 | 1.5 | 57.0 | 51.0 |
| ICU-2 | 2373 | 125.0 | 128 | 3.0 | 210.4 | 216.7 | 60.8 | 62 | 1.7 | 88.7 | 76.4 | 42.4 | 43 | 1.5 | 52.1 | 47.6 |
| ICU-3 | 1953 | 49.9 | 52 | 2.1 | 73.4 | 66.0 | 18.2 | 19 | 0.7 | 27.9 | 27.5 | 10.7 | 15 | 0.6 | 14.0 | 11.5 |
| ICU-4 | 1999 | 131.3 | 141 | 3.2 | 298.7 | 272.5 | 62.1 | 66 | 2.5 | 120.0 | 110.1 | 44.8 | 47 | 2.0 | 71.5 | 62.1 |
| ICU-5 | 3089 | 78.1 | 79 | 2.7 | 94.0 | 87.1 | 27.6 | 28 | 0.4 | 23.4 | 17.5 | 18.8 | 19 | 0.2 | 10.5 | 7.5 |
| ICU-6 | 2735 | 110.9 | 115 | 3.3 | 189.2 | 169.0 | 46.5 | 48 | 2.1 | 69.9 | 63.0 | 32.1 | 33 | 1.9 | 47.4 | 42.0 |
| ICU-7 | 1878 | 95.2 | 98 | 2.9 | 128.4 | 145.0 | 53.9 | 55 | 2.4 | 55.3 | 58.0 | 35.5 | 36 | 1.3 | 32.6 | 33.2 |

*indication of clustering of OTUs at various levels (0.01, 0.03, 0.05) using distance matrix.

Reads = number of good quality trimmed sequences per sample; Rf = rarefaction; OTU = operational taxonomic unit; H’ = Shannon diversity index; ACE = abundance-based coverage estimator; Chao1= non-parametric richness estimator.
