# Supplementary material for: Dogs Leaving the ICU Carry a Very Large Multi-Drug Resistant Enterococcal Population with Capacity for Biofilm Formation and Horizontal Gene Transfer
Source: PLoS One. 2011 Jul 19;6(7):e22451. doi: 10.1371/journal.pone.0022451 (PMC3139645; doi:10.1371/journal.pone.0022451)
Supplement: Table S3 — Distribution (%) of identified sequences from the feces of seven dogs from the intensive care unit (ICU) based on 454 pyrosequencing data of 16S rDNA. (DOC) [file pone.0022451.s003.doc]

**Table S3.** Distribution (%) of identified sequences from feces of seven dogs from the intensive care unit (ICU) based on 454 pyrosequencing data of 16S rDNA.

| **Phyum** | **Order/Family** | **Genus/Species** | | **ICU-1** | **ICU-2** | **ICU-3** | **ICU-4** | **ICU-5** | **ICU-6** | **ICU-7** |
| --- | --- | --- | --- | --- | --- | --- | --- | --- | --- | --- |
| ***Firmicutes*** | |  | |  |  |  |  |  |  |  |
|  | *Clostridia/Clostridiaceae* | *Sarcina* sp. | | 0.62 |  |  |  |  | 0.09 |  |
|  |  | *Clostridium celerecrescens* | |  |  |  |  |  |  | 0.06 |
|  |  | *Clostridium bolteae* | | 0.16 |  |  |  |  |  |  |
|  |  | *Clostridium difficile* | |  |  | 1.12 | 0.04 |  | 2.35 | 1.35 |
|  |  | *Clostridium symbiosum* | | 0.04 |  |  |  |  |  |  |
|  |  | *Clostridium glycolicum* | |  | 0.14 |  |  |  | 0.05 |  |
|  |  | *Clostridium perfringens* | | 0.62 |  |  | 1.61 |  |  |  |
|  |  | *Clostridium bartlettii* | | 0.29 |  |  | 0.30 |  | 0.14 |  |
|  |  | *Clostridium baratii* | | 0.12 |  |  |  |  |  |  |
|  |  | *Clostridium lituseburense* | |  |  |  | 0.04 |  |  |  |
|  |  | *Clostridium orbiscindens* | | 0.08 | 0.14 |  |  |  |  | 1.30 |
|  |  | *Clostridium* sp. | | 7.79 | 12.80 |  | 3.25 | 0.55 | 22.70 | 0.06 |
|  |  | *Clostridium sordellii* | | 0.12 |  |  |  |  |  |  |
|  | *Clostridia/Lachnospiraceae* | *Anaerostipes* sp. | | 0.04 |  |  |  |  |  | 0.23 |
|  |  | *Dorea* sp. | | 1.53 | 3.73 | 0.09 | 0.89 |  |  | 2.14 |
|  |  | *Roseburia* sp. | | 3.88 | 7.64 | 14.10 |  |  |  | 8.00 |
|  | *Clostridia/Ruminococcaceae* | *Ruminococcus* sp. | | 4.08 | 11.6 | 0.09 | 1.27 |  |  | 1.91 |
|  |  | *Anaerotruncus* sp. | | 0.33 |  |  |  |  |  |  |
|  |  | *Papillibacter* sp. | |  | 0.04 |  | 0.17 |  |  | 0.06 |
|  |  | *Subdoligranulum* sp. | | 0.12 | 0.04 |  |  |  |  |  |
|  |  | *Faecalibacterium* sp. | | 0.74 |  |  | 0.08 |  |  |  |
|  | *Clostridia/Enterobacteriaceae* | *Eubacterium* sp. | | 4.04 | 0.04 |  | 0.21 |  |  |  |
|  |  | *Eubacterium dolichum* | |  | 0.25 |  |  |  |  |  |
|  |  | *Eubacterium biforme* | | 1.65 |  |  |  | 0.06 |  |  |
|  | *Clostridia/Peptococcaceae* | *Peptococcus* sp. | | 0.04 |  |  |  |  |  |  |
|  | *Clostridia/Veillonellaceae* | *Megamonas* sp. | |  |  |  |  | 2.04 |  |  |
|  |  | *Dialister* sp. | | 0.04 |  |  |  |  |  |  |
|  | *Bacillaceae* | *Bacillus cereus* | |  |  |  |  | 0.03 |  |  |
|  |  | *Bacillus s*p. | |  |  |  |  | 0.03 |  |  |
|  | *Bacilli/Planococcaceae* | *Planomicrobium* sp. | |  |  |  |  |  |  | 0.06 |
|  | *Bacilli/Staphylococcaceae* | *Staphylococcus* sp. | |  |  | 0.04 | 0.04 |  |  |  |
|  | *Lactobacillaceae* | *Lactobacillus acidophilus* | | 43.00 |  |  | 1.06 |  |  |  |
|  |  | *Lactobacillus ruminis* | | 0.16 |  |  |  |  |  |  |
|  |  | *Lactobacillus* sp. | | 26.80 |  | 0.04 | 48.30 |  |  |  |
|  |  | *Lactobacillus reuteri* | |  |  |  | 1.14 |  |  |  |
|  | *Lactobacillales/Streptococcaceae* | *Streptococcus gallolyticus* | | 0.21 |  |  |  |  |  |  |
|  |  | *Streptococcus* sp. | |  |  |  | 0.04 |  |  |  |
|  |  | *Lactococcus* sp. | | 0.54 |  |  | 5.79 |  |  |  |
|  | *Lactobacillales/Enterococcaceae* | *Enterococcus* sp. | | 0.04 | 61.30 | 83.40 | 23.20 | 0.03 | 50.60 | 17.60 |
|  |  | *Enterococcus avium* | |  |  |  | 8.16 |  | 0.09 |  |
|  | *Lactobacillales/Carnobacteriaceae* | *Carnobacterium* sp. | |  |  |  |  |  | 0.09 |  |
|  | *Erysipelotrichaceae* | *Catenibacterium* sp. | | 0.41 |  |  |  |  |  |  |
|  |  | *Bulleidia* sp. | | 0.25 |  |  |  |  |  |  |
| ***Gammaproteobacteria*** | |  | |  |  |  |  |  |  |  |
|  | *Enterobacteriaceae* | *Citrobacter* sp. | |  | 0.83 |  |  |  |  |  |
|  |  | *Klebsiella* sp. | |  |  | 0.09 | 0.04 |  |  | 0.11 |
|  |  | *Escherichia* sp. | | 0.04 |  |  | 0.76 |  | 2.30 | 12.6 |
|  |  | *Escherichia coli* | | 0.16 |  |  | 2.32 |  | 1.36 | 15.8 |
|  |  | *Kluyvera* sp. | |  | 0.04 |  |  |  |  |  |
|  |  | *Grimontella* sp. | |  | 0.69 |  |  |  |  |  |
|  |  | *Enterobacter* sp. | |  |  |  | 0.04 |  |  |  |
|  |  | *Salmonella enterica* | |  |  |  |  |  | 0.05 |  |
|  |  | *Shigella boydii* | |  |  |  |  |  |  | 0.11 |
|  |  | *Shigella* sp. | | 0.08 |  |  | 0.72 |  | 9.07 | 19.10 |
|  | *Moraxellaceae* | *Psychrobacter* sp. | |  |  |  |  |  | 2.21 |  |
|  |  | *Acinetobacter* sp. | |  |  |  |  |  | 1.55 |  |
|  | *Brucellaceae* | *Ochrobactrum* sp. | |  | 0.07 |  |  |  |  |  |
| ***Alphaproteobacteria*** | |  | |  |  |  |  |  |  |  |
| *Rhizobiales/Methylobactericeae* | | *Methylobacterium* sp. | |  | 0.07 |  |  |  |  |  |
| ***Actinobacteria*** | |  | |  |  |  |  |  |  |  |
|  | *Coriobacteriaceae* | *Collinsella* sp. | | 1.86 | 0.36 | 0.81 |  | 0.03 |  | 0.62 |
|  |  | *Slackia* sp. | |  |  |  |  | 0.03 |  |  |
|  |  | *Eggerthella* sp. | | 0.08 |  |  |  |  |  |  |
|  | *Microbacteriaceae* | *Frigoribacterium* sp. | |  |  |  |  |  | 0.05 |  |
|  | *Alicrococcaceae* | *Arthrobacter* sp. | |  |  |  |  |  | 7.15 |  |
|  |  | *Arthrobacter stackebrandtii* | | |  |  |  |  | 0.05 |  |
|  | *Corynebacteriaceae* | *Corynebacterium* sp. |  | |  |  | 0.25 | 0.06 |  |  |
|  | *Corynebacterinae/Nocardiaceae* | *Rhodococcus* sp. |  | |  |  |  |  | 0.09 |  |
|  |  | *Friedmanniella* sp. |  | | 0.04 |  |  |  |  |  |
| ***Bacteroidetes*** | |  |  | |  |  |  |  |  |  |
|  | *Bacteroidaceae* | *Bacteroides massiliensis* |  | |  |  |  |  |  | 0.17 |
|  |  | *Bacteroides vulgatus* |  | |  |  |  |  |  | 8.00 |
|  |  | *Bacteroides* sp. |  | | 0.11 |  |  | 5.60 | 0.05 | 10.3 |
|  |  | *Bacteroides coprocola* |  | |  |  |  | 0.46 |  |  |
|  | *Bacteroidales/Rikenellaceae* | *Rikenella* sp. |  | |  |  |  | 0.03 |  |  |
|  | *Bacteroidales/Privotellaceae* | *Prevotella* sp. |  | |  | 0.18 |  |  |  |  |
| ***Fusobacteria*** | |  |  | |  |  |  |  |  |  |
|  | *Fusobacteriaceae* | *Fusobacterium* sp. |  | | 0.04 |  | 0.21 | 91.10 |  | 0.45 |
| ***Mollicutes*** | |  |  | |  |  |  |  |  |  |
|  | *Aneroplasmataceae* | *Anaeroplasma* sp. |  | |  |  |  | 0.03 |  |  |
