# Supplementary material for: Dogs Leaving the ICU Carry a Very Large Multi-Drug Resistant Enterococcal Population with Capacity for Biofilm Formation and Horizontal Gene Transfer
Source: PLoS One. 2011 Jul 19;6(7):e22451. doi: 10.1371/journal.pone.0022451 (PMC3139645; doi:10.1371/journal.pone.0022451)
Supplement: Table S4 — Multiple (≥3) antibiotic resistance profile among enterococci from the feces of dogs from the intensive care unit (ICU). (DOC) [file pone.0022451.s004.doc]

**Table S4.** Multiple (≥ 3) antibiotic resistance profile among enterococci from the feces of dogs­ from the intensive care unit (ICU).

| **Resistance profile** |  | ***E. faecalis* (n=94)** |  | ***E. faecium* (n=113)** |
| --- | --- | --- | --- | --- |
|  |  | **No. of strains (%)** |  | **No. of strains (%)** |
| AM, TET, ENO |  |  |  | 13 (11.5) |
| AM, D, TET |  | 1 (1.1) |  |  |
| AM, STR, ENO |  |  |  | 5 (4.4) |
| AM, ENO, NF |  |  |  | 4 (3.5) |
| TET, D, ENO |  | 11 (11.7) |  |  |
| TET, E, ENO |  |  |  | 1 (0.9) |
| TET, D, E |  | 23 (24.5) |  |  |
| TET, D, E, ENO |  | 6 (6.4) |  |  |
| AM, TET, ENO, NF |  |  |  | 1 (0.9) |
| AM, STR, ENO, NF |  |  |  | 4 (3.5) |
| AM, TET, STR, ENO |  |  |  | 6 (5.3) |
| AM, TET, GM, ENO |  |  |  | 1 (0.9) |
| AM, TET, D, ENO |  |  |  | 2 (1.8) |
| AM, TET, D, ENO, NF |  |  |  | 1 (0.9) |
| AM, TET, GM, STR, ENO |  |  |  | 1 (0.9) |
| AM, TET, D, E, ENO |  | 1 (1.1) |  | 6 (5.3) |
| AM, GM, STR, E, ENO |  |  |  | 3 (2.6) |
| AM, TET, D, STR, ENO |  |  |  | 5 (4.4) |
| AM, TET, D, E, ENO, NF |  |  |  | 2 (1.8) |
| AM, TET, GM, STR, E, ENO |  |  |  | 1 (0.9) |
| AM, TET, D, STR, ENO, NF |  |  |  | 1 (0.9) |
| AM, TET, D, GM, E, ENO |  |  |  | 25 (22.1) |
| AM, TET, D, GM, E, ENO, NF |  |  |  | 4 (3.5) |
| AM, TET, D, GM, STR, E, ENO |  |  |  | 8 (7.0) |
| AM, TET, D, GM, STR, ENO, NF |  |  |  | 1 (0.9) |
| AM, TET, D, GM, STR, E, ENO, NF |  |  |  | 7 (6.2) |

**Abbr.** AM = ampicillin, TET = tetracycline, D = doxycycline, GM = gentamicin, STR = streptomycin, E = erythromycin, ENO = enrofloxacin, NF = nitrofurantoin.
