# Supplementary material for: Dogs Leaving the ICU Carry a Very Large Multi-Drug Resistant Enterococcal Population with Capacity for Biofilm Formation and Horizontal Gene Transfer
Source: PLoS One. 2011 Jul 19;6(7):e22451. doi: 10.1371/journal.pone.0022451 (PMC3139645; doi:10.1371/journal.pone.0022451)
Supplement: Table S5 — (A) Antibiotic resistance phenotype and virulence genotypic profile of E. faecalis from individual ICU dogs. (B) Antibiotic resistance phenotype, virulence genotypic profile, and MLVA types (MTs) of E. faecium from individual ICU dogs. Isolates are grouped (color coded) based on their antibiogram. ICU = intensive care unit, R = resistance to antibiotics, ‘+’ = presence of virulence gene, NT = not typeable. (PDF) [file pone.0022451.s005.pdf]

Table S5. **(A) Antibiotic resistance phenotype and virulence genotypic profile of *E. faecalis* from individual ICU dogs.**
(B) Antibiotic resistance phenotype, virulence genotypic profile, and MLVA types (MTs) of *E. faecium* from individual ICU dogs.
Isolates are grouped (color coded) based on their antibiogram.
ICU = intensive care unit, R = resistance to antibiotic, '+' = presence of the virulence gene.

A. *E. faecalis*

	TGC	NF	ENO	AM	E	D	GM	VA	TET	STR	LIN	*gelE*	*cylA*	*esp*	*asa1*
**ICU-1**															
**2**					R	R			R			+		+	
**8**					R	R			R				+	+	+
**16**					R	R			R			+	+	+	+
**21**					R	R			R				+	+	+
**25**					R	R			R				+	+	+
**26**					R	R			R				+	+	+
**27**					R	R			R				+	+	+
**28**					R	R			R				+	+	+
**29**					R	R			R				+	+	+
**11**					R	R			R			+		+	+
**12**					R	R			R			+		+	+
**20**					R	R			R			+		+	+
**13**					R	R			R					+	+
**15**					R	R			R					+	+
**17**					R	R			R					+	+
**18**					R	R			R					+	+
**23**					R	R			R					+	+
**6**					R	R			R					+	+
**14**					R	R			R					+	+
**19**					R	R			R					+	+
**22**					R	R			R					+	+
**5**					R				R					+	+
**9**					R				R					+	+
**10**					R				R					+	+
**30**					R				R					+	+
**1**						R			R			+		+	
**3**						R			R			+		+	
**4**				R					R			+			
**7**			R		R	R			R				+	+	+

**24**	R			R	R	+		+	+

**ICU-2**									
**2**			R			+			
**3**			R			+			
**9**			R			+			
**11**			R			+			
**17**			R			+			
**23**			R			+			
**1**	R		R			+			
**4**	R					+			
**24**		R	R			+			
**15**						+			

**ICU-3**									
**12**						+	+		
**13**						+	+		
**15**						+	+		
**23**						+	+		
**27**						+	+		
**28**						+	+		
**14**			R			+	+		
**22**			R			+	+		
**24**			R			+	+		
**25**			R			+	+		
**26**			R			+	+		
**11**			R		R	+			

**ICU-4**									
**1**	R			R	R	+			
**46**	R			R	R	+			
**52**	R			R	R	+			
**9**	R			R	R	+			
**42**	R			R	R	+		+	
**57**	R			R	R	+		+	
**59**	R			R	R	+			
**60**	R			R	R	+		+	
**18**	R			R	R	+			
**24**	R			R	R	+			

29			R			R			R			+		
64						R			R			+		
25						R			R			+		
26						R			R			+		
55						R			R			+		+
14						R			R			+		+
15						R			R			+		
27						R			R			+	+	
2			R		R	R			R			+		
28			R		R	R			R			+		
30			R		R	R			R			+		
45					R	R			R			+		
54					R	R			R			+		
13			R	R	R	R			R			+		
62				R		R			R			+		

ICU-7

1			R									+		
22			R									+		
23			R									+		
24			R									+		
30			R									+		
3			R		R							+		
4			R		R							+		
5			R		R							+		
14			R		R							+		
21			R		R							+		
25			R		R							+		
6												+		
12												+		
13												+		
31												+		
15												+		
11					R							+		

B. ***E. faecium***

	TGC	NF	Q/D	ENO	AM	E	D	GM	VA	TET	STR	LIN	*gelE*	*cylA*	*esp*	*asa1*	MT
**ICU-2**																	

**5**		R	R	R	R		R		+		**10**
**7**		R	R	R	R		R		+		**10**
**20**		R	R	R	R		R		+		**27**
**10**		R	R	R	R	R	R	R	+		**12**
**25**		R	R	R	R	R	R	R	+		**338**
**29**		R	R	R	R	R	R	R	+		**12**
**30**		R	R	R	R	R	R	R	+		**337**
**6**	R	R	R	R	R	R	R	R	+		**338**
**13**	R	R	R	R	R	R	R	R	+		**337**
**16**		R	R	R	R	R	R		+		**12**
**22**		R	R	R	R	R	R		+		**12**
**26**		R	R	R		R		R	+		**337**
**28**		R	R	R		R		R	+		**338**
**14**		R	R	R		R		R	+		**338**
**8**	R	R	R	R	R		R		+		**12**
**12**	R	R	R			R	R	R	+		**27**
**19**							R		+		**338**
**21**		R	R					R	+		**27**
**27**		R	R			R	R	R	+		**27**

ICU-3

**1**		R	R	R	R	R	R	+	+	**1**
**2**		R	R	R	R	R	R	+	+	**1**
**3**		R	R	R	R	R	R	+	+	**1**
**4**		R	R	R	R	R	R	+	+	**1**
**5**		R	R	R	R	R	R	+	+	**1**
**8**		R	R	R	R	R	R	+	+	**1**
**10**		R	R	R	R	R	R	+	+	**1**
**17**		R	R	R	R	R	R	+	+	**1**
**19**		R	R	R	R	R	R	+	+	**1**
**20**		R	R	R	R	R	R	+	+	**1**
**29**		R	R	R	R	R	R	+	+	**1**
**30**		R	R	R	R	R	R	+	+	**1**
**51**		R	R	R	R	R	R	+	+	**1**
**52**		R	R	R	R	R	R	+	+	**1**
**9**	R	R	R	R	R	R	R	+	+	**1**
**16**	R	R	R	R	R	R	R	+	+	**1**
**21**	R	R	R	R	R	R	R	+	+	**1**
**49**	R	R	R	R	R	R	R	+	+	**1**

ICU-4

23		R	R			R	R		+		27
12		R		R			R				NT
21				R			R				NT
41				R			R				NT

ICU-5

4		R	R				R		+		335
5		R	R				R		+	+	335
7		R	R				R		+	+	335
9		R	R				R				335
10		R	R				R		+	+	335
13		R	R				R		+	+	335
14		R	R				R		+	+	335
20		R	R				R		+	+	335
23		R	R				R		+	+	335
24		R	R				R				335
25		R	R				R		+	+	335
27		R	R				R		+	+	335
29		R	R				R		+	+	335
2		R	R				R	R	+	+	335
6		R	R				R	R	+	+	335
8		R	R				R	R	+	+	335
16		R	R				R	R	+	+	335
17		R	R				R	R	+	+	335
28		R	R				R	R	+	+	335
22		R	R		R		R	R	+	+	335
11		R	R		R		R	R	+	+	335
15		R	R		R		R	R	+	+	335
18		R	R		R		R	R	+	+	335
21		R	R		R		R	R	+	+	335
1		R	R		R		R		+		335
12		R	R		R		R		+	+	335
3	R	R	R				R		+		335
19	R	R	R		R		R	R	+	+	335
26	R	R	R		R		R		+	+	335

ICU-6

8	R	R	R	R	R	R	R	R	+		30

**9**	R	R	R	R	R	R	R	R	+	**30**
**11**	R	R	R	R	R	R	R	R		**30**
**12**	R	R	R	R	R	R	R	R		**30**
**16**	R	R	R	R	R	R	R	R	+	**30**
**2**		R	R	R	R	R	R	R	+	**30**
**21**		R	R	R	R	R	R	R		**30**
**22**		R	R	R	R	R	R	R		**30**
**27**		R	R	R	R	R	R	R	+	**30**
**1**		R	R					R	+	**336**
**3**		R	R					R	+	**336**
**7**		R	R					R	+	**336**
**26**		R	R					R		**336**
**14**		R	R						+	**336**
**15**		R	R							**336**
**20**		R	R							**336**
**29**		R	R						+	**336**
**4**		R	R		R	R	R	R	+	**30**
**18**		R	R		R	R	R	R	+	**30**
**23**		R	R		R	R	R	R	+	**30**
**30**		R	R		R	R	R	R	+	**30**
**5**	R	R	R					R	+	**336**
**10**	R	R	R					R		**336**
**13**	R	R	R					R		**336**
**19**	R	R	R					R		**336**
**6**	R	R	R	R	R	R	R	R	+	**30**
**25**	R	R	R	R	R	R	R	R	+	**30**
**17**	R	R	R						+	**336**
**24**	R	R	R							**336**
**28**	R	R	R		R	R	R	R	+	**30**

ICU-7

**7**		R	R	R	R	R	R	+	**1**
**10**		R	R	R	R	R	R	+	**1**
**17**		R	R	R	R	R	R	+	**10**
**20**		R	R	R	R	R	R	+	**1**
**28**		R	R	R	R	R	R	+	**1**
**19**		R	R	R	R		R	+	**10**
**26**		R	R	R	R		R	+	**10**
**27**		R	R	R	R		R	+	**10**
**8**	R	R	R	R	R		R	+	**10**

**29**	R		R	R	R	R			R			+	**10**
**16**	R		R	R	R	R			R			+	**10**
**9**	R		R	R	R	R	R		R			+	**10**
**18**	R		R	R	R	R	R		R			+	**1**

Abbr. AM, ampicillin; TET, tetracycline; D, doxycycline; GM, gentamicin; STR, streptomycin;
E, erythromycin; ENO, enrofloxacin; VA, vancomycin; Q/D, quinupristin/dalfopristin; LIN, linezolid; NF, nitrofurantoin;
TGC, tigecycline.
